# Extracellular vesicle expansion of *PMIS‐miR‐210* expression inhibits colorectal tumour growth via apoptosis and an XIST/NME1 regulatory mechanism

**DOI:** 10.1002/ctm2.1037

**Published:** 2022-09-18

**Authors:** Steven Eliason, Liu Hong, Yan Sweat, Camille Chalkley, Huojun Cao, Qi Liu, Hank Qi, Hongwei Xu, Fenghuang Zhan, Brad A. Amendt

**Affiliations:** ^1^ Department of Anatomy and Cell Biology The University of Iowa Iowa City Iowa USA; ^2^ Craniofacial Anomalies Research Center The University of Iowa Iowa City Iowa USA; ^3^ Iowa Institute for Oral Health Research The University of Iowa Iowa City Iowa USA; ^4^ Department of Internal Medicine University of Arkansas for Medical Science Little Rock Arkansas USA

**Keywords:** colorectal cancer, microRNA mouse models, microRNA therapeutic, miR‐210, NME1, plasmid‐based microRNA inhibitor system (PMIS), XIST

## Abstract

**Background:**

Colorectal cancer (CRC) has a high mortality rate, and therapeutic approaches to treat these cancers are varied and depend on the metabolic state of the tumour. Profiles of CRC tumours have identified several biomarkers, including microRNAs. *microRNA‐210* (*miR‐210)* levels are directly correlated with CRC survival. *miR‐210* expression is higher in metastatic colon cancer cells versus non‐metastatic and normal colon epithelium. Therefore, efficient methods to inhibit *miR‐210* expression in CRC may provide new advances in treatments.

**Methods:**

Expression of miRs was determined in several metastatic and non‐metastatic cell lines. *miR‐210* expression was inhibited using *PMIS‐miR‐210* in transduced cells, which were transplanted into xenograft mice. In separate experiments, CRC tumours were allowed to grow in xenograft mice and treated with therapeutic injections of *PMIS‐miR‐210*. Molecular and biochemical experiments identified several new pathways targeted by *miR‐210* inhibition.

**Results:**

*miR‐210* inhibition can significantly reduce tumour growth of implanted colon cancer cells in xenograft mouse models. The direct administration of *PMIS‐miR‐210* to existing tumours can inhibit tumour growth in both *NSG* and *Foxn1^nu/j^* mouse models and is more efficacious than capecitabine treatments. Tumour cells further transfer the *PMIS‐miR‐210* inhibitor to neighbouring cells by extracellular vesicles to inhibit *miR‐210* throughout the tumour. *miR‐210* inhibition activates the cleaved caspase 3 apoptotic pathway to reduce tumour formation. We demonstrate that the long non‐coding transcript *XIST* is regulated by *miR‐210* correlating with decreased *XIST* expression in CRC tumours. *XIST* acts as a competing endogenous RNA for *miR‐210*, which reduces *XIST* levels and *miR‐210* inhibition increases *XIST* transcripts in the nucleus and cytoplasm. The increased expression of *NME1* is associated with H3K4me3 and H3K27ac modifications in the *NME1* proximal promoter by *XIST*.

**Conclusion:**

Direct application of the *PMIS‐miR‐210* inhibitor to growing tumours may be an effective colorectal cancer therapeutic.

## Graphical abstract

- A new 120 nt microRNA inhibitor therapeutic is specific, stable, efficient and non‐toxic to cells
- Injection of the inhibitor into growing colorectal tumors inhibits and reduces tumor growth.
- *miR‐210* inhibition activates apoptosis, *XIST* and NME1 to reduce tumor growth
- This approach highlights the therapeutic application of the plasmid‐based microRNA inhibition system (PMIS) for treating tumors.

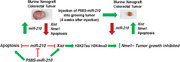

## 1 INTRODUCTION

Colorectal cancer (CRC) is the third most prevalent type of cancer worldwide and has one of the highest mortality rates of any cancer, with 1.3 million new cases of CRC diagnosed worldwide every year and 700 000 deaths per year.^1^ Tumour progression and metastasis of CRC are complex multifactorial processes that are not fully understood. Despite advances in personalized medicine, chemotherapy and immune therapies, CRC patients still have reoccurrences at a significant rate and the mortality rate remains significant.^2^, ^3^

microRNAs (miRs) are small non‐coding RNA molecules that regulate expression primarily by binding messenger RNA and inhibiting translation, often, but not exclusively at the 3′UTR. miRs have also been shown to bind to the 5′ enhancer/promoters of genes.^4^, ^5^, ^6^ miRs have been implicated in many developmental and biological processes and have been shown to be responsible for tumour progression in several tumour types.^7^, ^8^, ^9^ Many methods to inhibit miRs have been described, including antisense oligos, antagomirs and LNAs (locked nucleic acids) technologies.^10^, ^11^, ^12^, ^13^, ^14^ Oligonucleotides can be modified to increase their stability in cells, but must be continually applied to dividing cells. Oligonucleotides such as antagomirs and LNA must be delivered to cells using toxic and expensive delivery systems.

The plasmid‐based microRNA inhibitor system (PMIS) is an miR inhibition system that comprises a 122nt miR binding structure designed to interact with RNA‐induced silencing complex (RISC) factors to load the mature miR into a stable inhibitor complex with high efficiency, specificity and stability.^15^, ^16^ This inhibitor is expressed from a plasmid or construct without chemical modifications that produce continual expression of a specific antisense RNA molecule in cells and transgenic mice.^15^, ^16^, ^17^, ^18^, ^19^, ^20^ The PMIS delivers physiological levels of specific miR inhibitors to cells and in transgenic mice with high specificity and no off‐target effects or toxicity.^15^, ^16^

Because increased *miR‐210* levels are directly correlated with metastatic colorectal tumours, we hypothesized that using *PMIS‐miR‐210* would effectively decrease tumour growth. *miR‐210* is expressed in many cell types and regulates multiple biological processes, including the aetiology of several cancer progressions.^21^, ^22^, ^23^ High levels of *miR‐210* have been implicated in lung, prostate, oesophageal and bladder cancer, and predict poor survival in cancer patients.^24^, ^25^ *miR‐210* is regulated by hypoxia and helps regulate the hypoxia response and HIF‐1a binds to a hypoxia response element in the *miR‐210* proximal promoter.^26^, ^27^ Because *miR‐210* can target many processes including angiogenesis, apoptosis, DNA repair, cell cycle progression and mitochondrial function, it is considered an exceptional therapeutic target.^26^ Furthermore, *miR‐210* levels correlate with tumour prognosis, and individuals with CRC have a poor long‐term prognosis if the initial tumour is *miR‐210* high versus *miR‐210* low.^28^

We profiled *PMIS‐miR‐210* transduced and untreated CRC cells and tumours for gene expression changes and focused on two genes *XIST* and *NME1* that were significantly affected by *miR‐210* inhibition. *XIST* is a long non‐coding RNA that is involved in sex‐specific inactivation of one X chromosome in females. The mis‐regulation of *XIST* has been reported in numerous cancers. *XIST* may have tumour suppressor functions in cervical, breast, hepatocellular, prostate and osteosarcoma cancers^29^, ^30^, ^31^, ^32^, ^33^, ^34^ and may act as a tumour promoter in pancreatic, bladder, colon, thyroid, gastric and glioblastoma cancers.^35^, ^36^, ^37^, ^38^, ^39^, ^40^, ^41^ *NME1* is a nucleoside diphosphate kinase, and *NME* family members share significant homology, including some members with DNA binding domains that regulate transcription and DNA repair. *NME1* can regulate gene expression by binding to promoter/enhancer regions of genes that contribute to tumour metastasis suppressor function.^42^

In this study, we first generated stable colon cancer cell lines expressing *PMIS*‐empty vector (EV) control or *PMIS‐miR‐210* and performed in vitro assays to identify morphology, growth and gene expression changes, and use an explant model to test in vivo tumour growth of these lines. Furthermore, we test the ability of *PMIS‐miR‐210* injections at inhibiting the growth of existing tumours. *PMIS‐miR‐210* transcripts are found in extracellular vesicles (ECVs) and these ECVs allow for the spread of *PMIS‐miR‐210* expression within the tumour. The inhibition of *miR‐210* in cells changes several key in vitro characteristics and causes a significant reduction of tumour growth in xenograft mice. *miR‐210* can bind and regulate *XIST* transcripts and *miR‐210* inhibition increases *XIST* transcripts and *XIST* acts to modulate epigenetic factors. We show that the inhibition of *miR‐210* caused an increase in H3K4me3 and H3K27ac chromatin deposition at the *NME1* promoter associated with an upregulation of *NME1*. *XIST* transcripts are stabilized by the loss of *miR‐210* and appear to induce these transcriptional active marks on the *NME1* proximal promoter.

## 2 MATERIALS AND METHODS

### 2.1 Study design

#### 2.1.1 Sample size

Predetermined sample size was estimated from statistical analyses and also to demonstrate effectiveness of the experimental procedure. A general analysis was based on pilot studies and performed based on Lehr's formula with one outcome and analysis used was the student's *t*‐test. We added additional samples (another immunodeficient mouse line) to confirm the previous results.

#### 2.1.2 Rules for stopping data collection

Final endpoints for tumour size and shape were determined by the University of Iowa office of animal care and all procedures were approved by the Institutional Animal Care and Use Committee (IACUC). Once tumours reached a predetermined size, animal care informed us to stop the study.

#### 2.1.3 Data inclusion/exclusion criteria

All mouse data are included in the manuscript, no mice died or were excluded from the data. We report all data, nothing was omitted.

#### 2.1.4 Selection of endpoints

The growing tumour size determined the endpoint of the study. Once tumours reached a measurable size, treatment began and all mice responded to the treatment and no mice were excluded from the study. Tumour size was dependent on the amount of cells injected into the flanks of mice and determined with caliper and bioluminescence imaging (BLI) imaging analyses.

#### 2.1.5 Replicates

Each experiment was performed between four and six times. Replicates were determined after a set number of mice were treated. All results were substantiated by each repetition.

#### 2.1.6 Research objectives

Because colorectal cancers and tumour profiles showed that patients with high *miR‐210* expression had a low survival rate, our hypothesis stated that ‘if we use our specific *PMIS‐miR‐210* inhibitor, it would effectively inhibit tumour growth’. Our hypothesis was validated after initial mouse experiments.

#### 2.1.7 Research subjects

Two types of immunodeficient mice models were used in the study to validate our system. Furthermore, we not only used *PMIS‐miR‐210*‐transduced colorectal tumour cells before injection into the flanks of the mice and measured tumour formation, we also let the tumours grow and then treated them with direct injection of *PMIS‐miR‐210* plasmid DNA.

#### 2.1.8 Experimental design

Mice were randomly injected with different concentrations of colorectal tumour cells, and treatments were applied in a blinded study before images of the tumours (BLI before treatment) were analyzed. Mice were randomly selected for different treatments without prior knowledge of tumour size or shape.

#### 2.1.9 Randomization

All mice were treated identically and no mice were excluded.

#### 2.1.10 Blinding

Prior to injection of *PMIS‐miR‐210* or controls into the tumours, the mice were randomly selected and no preference was given to a mouse tumour size and shape by BLI imaging prior to treatments.

#### 2.1.11 Statistical analysis

All experiments were independent replicates of four to six.

### 2.2 Cell culture, transient transfections, luciferase and β‐galactosidase assays

The CCD841 (normal colon epithelium), SW480 (primary colon tumour), SW620 (secondary/metastatic tumour from same patient as SW480), Colo320, DLD1 (colon cancer cell lines) and HEK 293 cells were obtained from ATCC and cultured in DMEM supplemented with 10% FBS and penicillin/streptomycin. Briefly, according to ATCC, SW480 cells are from a primary CRC tumour and SW620 is from a CRC tumour that has metastasized to the lymph node, same patient as SW480. Transfected cells were incubated for 48 h and then lysed for reporter activities and protein content by Bradford assay (Bio‐Rad) as previously described.^43^, ^44^ Western blots were performed as previously described.^43^, ^44^ ECVs were isolated from the media of cells cultured for 24 h using the miRCURY cell/urine/CSF kit (Qiagen).

Transwell plates using 3.0 μm pore sizes were used (Nunc, Thermo Fisher). Transwell experiments were performed by plating 2 × 10^5^ recipient SW620 cells into six‐well plates. Within the transwell insert, 0.5, 1 or 2 × 10^5^ donor cells were seeded, SW620 *PMIS‐miR‐210*. After 72 h, the inserts were removed and RNA was isolated from the recipient cells using the RNA easy miR kit (Qiagen). The EV inhibitor Neticonazole (Selleck chemicals) was used at 10 μm in culture for 48 h. Control exosome preps and RNA were isolated from SW620 and SW620 *PMIS‐miR‐210* cultures with 10 μm neticonazole to demonstrate the effectiveness of the EV inhibitor in these cells. All experiments were repeated three times.

### 2.3 PMIS lentiviral vector generation, plasmids and cell proliferation assays

The plasmid‐based microRNA inhibition system (PMIS system; https://naturemiri.com/) was previously described.^15^, ^16^ The PMIS molecule is 122 nucleotides containing an antisense sequence to a specific miR in a stem loop. It has a high affinity for the mature miR and interacts with the RISC complex. The PMIS can distinguish and selectively inhibit miRs with only one nucleotide difference. A second‐generation lentiviral vector system was used to transduce SW620 cells to generate stable lines expressing *PMIS‐miR‐210* and *PMIS‐EV*. The packaging vectors were co‐transected with psPAX2 and pMDG.2 (Addgene) into HEK 293 FT cells to generate lentiviral molecules. Stable cell lines were generated and maintained in puro selection at 0.5ug/ml. A luciferase positive line was generated using LV570 plasmids to generate luciferase positive SW620 cells (Genetarget, Inc). Transfections were conducted using PEI‐mediated DNA introduction into cells, including *XIST* expression, *pXIST*, *NME* expression (Addgene) and *miR‐210* over expression plasmids.

### 2.4 Plasmid DNA preparations

All plasmid preps were generated by alkaline lysis followed by CsCl gradient purification, double banded CsCL purified as done previously.^44^, ^45^

### 2.5 RNA and qPCR analysis

Total RNA, including PMIS lines from cells and from tumour tissue, was prepared using the trizol (Thermo Fisher) and miRNeasy Mini Kit when required (Qiagen). Multiple isolations were performed, and separate qPCR assays were run on each sample (*N* = 3). The amount and integrity of the RNA samples were assessed by measurement at 260 and 280 nm and by agarose gel analyses. Quantitative real‐time PCR for mature miR expression was done with Qiagen microRNA assay system (Qiagen, CA, USA), including U6B as a reference gene. Total RNA was reverse transcribed into cDNA using reverse transcriptase and mix of OligoDT and random primers. Real‐time PCR was carried out using SYBR Green Supermix, 0.1 μmol/L forward primer, 0.1 μmol/L reverse primer and 0.25 μl cDNA template in a Bio‐Rad cycler (Takara, CA, USA). β‐tubulin served as a reference gene and ΔΔ*C*_t_ values were used to calculate fold differences. Melting curve analyses were performed to confirm amplification specificity of the PCR products and each probe was initially sequenced. PCR primers are listed in Table S1. miR levels were assessed using the micro scriptRT system (Qiagen) and miR‐specific primers and qPCR.

### 2.6 RNA sequencing

RNA sequencing was performed using SW620 *PMIS‐EV* and SW620 *PMIS‐miR‐210* cell lines, and tumours generated from those cell lines by LC sciences (Houston, TX, USA) and analyzed using TruSeq Stranded RNA‐seq software. Using the Illumina paired‐end RNA‐seq approach, we sequenced the transcriptome, generating a total of millon 2 × 150 bp paired‐end reads. Reads obtained from the sequencing machines include raw reads containing adapters or low quality bases, which will affect the following assembly and analysis. Thus, to get high‐quality clean reads, reads were further filtered by Cutadapt (https://cutadapt.readthedocs.io/en/stable/, version: cutadapt‐1.9). Genes with a two‐fold or greater expression change in PMIS‐210 versus PMIS‐EV were compared among the cells and the tumours and 120 genes showed similar expression changes. Pathway analysis was conducted using functional gene sets, gene ontology (GO) and gene set enrichment analysis (GSEA). Putative targets were verified by qPCR, and the 3′UTRs were cloned for *NME1* and *FGFRL1* into the pMIR‐luciferase reporter and tested for activity with and without inhibition by *miR‐210*. Statistical significance was determined using the Student's *t*‐test.

### 2.7 Xenograft model

All mouse studies were done under the guidelines of the University of Iowa office of animal care and all procedures were approved by the IACUC committee. Male nude mice (*Foxn1^nu/j^*; ages 6–7 weeks, JAX stock #002019) and *NSG* (NSG ages 6–7 weeks, JAX stock strain #005557) mice were purchased from Jackson labs and were maintained in laminar flow cages under pathogen‐free conditions. The selection of dose, amount of cells and schedule of treatments in mice were calculated based on previous experiments, cell growth to tumour formation and treatments were set when tumour growth reached effective size for treatments. Furthermore, the University of Iowa office of animal care put restrictions on allowable tumour sizes, so treatments began before tumours were considered too large for the animals. 5 × 10^5^ SW620 *PMIS‐EV* cells or SW620 *PMIS‐miR‐210* cells were suspended in sterile saline and were administered by subcutaneous injection into either flank area of nude mice. The mice were weighed, and tumour sizes were measured every other day with calipers for calculation of tumour size, length and width. Tumours were harvested after 35–40 days.

Mice were analyzed by BLI at 7–10 days after SW620 cell implantation to show that each flank received tumours and again at 35–40 days after implantation, mice were then sacrificed, and the tumours were collected and weighed. For some experiments, the tumours were dissected into two equal halves, one for RNA and one placed in 4% PFA for tissue analysis. For RNA extraction, tumour pieces were flash‐frozen in LN2, tissue was crushed and lysed in trizol, and RNA was extracted using the Qiagen miRNeasy Extraction Kit (Qiagen). Analysis of the expression levels of miR‐210 and other control or target genes was performed by qPCR as described earlier.

In separate studies, luciferase‐positive SW620 (1 × 10^6^ cells, SW620 LV+) were injected into both flanks of 6–7‐week‐old *Foxn1^nu/j^* or *NSG* mice and the tumours were allowed to grow for 7–10 days, until clearly visible under BLI conditions to determine localization and size of the injected tumour. Naked plasmid DNA (*PMIS‐miR‐210*, *PMIS‐EV* or no DNA/normal saline) was injected directly into and around each tumour. DNA in sterile saline was suspended at 50 or 100 ng/μl and 100 μl was injected. 100 μl (5 or 10 μg) was injected into the solid mass of the tumour at days 7–10 and then again after 5 days. Several mice with tumours implanted were given capecitabine (Sigma) treatment, a known colon cancer drug at a dose of 750 mg/kg by gavage oral administration. Caliper measurements were made at the time of the DNA injection prior to treatments and after treatments. 35–40 days after tumour formation, tumours were imaged by BLI methods and removed and processed as mentioned previously.

### 2.8 Aspartate transferase and alanine aminotransferase measurements

Serum from mice was measured for aspartate transferase (AST) and alanine aminotransferase (ALT) levels using the manufacturer‐recommended conditions (Sigma). One unit of AST is the amount of enzyme that will generate 1.0 mole of glutamate per minute at pH 8.0 at 37°C. One unit of ALT is defined as the amount of enzyme that generates 1.0 μmol of pyruvate per minute at 37°C.

### 2.9 Bioluminescence imaging

To image tumour cells in vivo, mice were i.p. injected with 100 μl of luciferin stocks (in sterile saline). The animals were anaesthetized with isoflurane (2% in 1 L/min oxygen), and bioluminescence images (BLI) were acquired using the IVIS Lumina system (a Xenogen Product from Caliper Life Sciences, now Perkin‐Elmer). Images were acquired every 2 min (10 s exposure/image).

### 2.10 Haematoxylin and eosin and IHC

Tumours were fixed in 4% PFA, dehydrated and embedded in paraffin. Sections were stained in haematoxylin and eosin (H&E) or treated for antigen retrieval and stained for IF with Ki67 (Abcam), GFP (Invitrogen), Caspase C3 (DSHB, Iowa), NME1 (Thermo Fisher), CD9 and CD31 (DSHB, Iowa) antibodies.

### 2.11 H3K4me3 and H3K27ac ChIP‐qPCR

Chromatin immunoprecipitations (ChIPs) were performed as described here. Formaldehyde‐cross‐linked cells were lysed and sonicated to shear the DNA. The sonicated DNA–protein complexes were immunoprecipitated (IP) with the following antibodies: control IgG (A01008, GenScript), anti‐H3K4me3 (ab8580, Abcam) and anti‐H3K27ac (ab4729, Abcam). The immuno‐complexes were collected using protein A/G agarose beads. The eluted DNA and 10% of respective input DNA were reverse cross‐linked at 65°C overnight and used for the qPCR using SYBR Green qPCR mix and a CFX96 instrument (BioRad). The enrichments were calculated as percentage of input. Folds change over input was displayed after normalizing to UTR region.

### 2.12 *XIST* in situ experiments

SW620 or SW620 *PMIS‐miR‐210* cells were seeded on glass cover slips and allowed to grow for 24 h. Cells were fixed and permeabilized under RNAase‐free conditions and in situ hybridization was performed using tellaris FISH in situ probes labelled with Q570 (Biosearch Technologies) for 16 h at 55°C, washed, counterstained with DAPi and imaged using the Confocal Zeiss 700.

### 2.13 Extracellular vesicle isolation and identification

Extracellular vesicles were isolated from the media of cells cultured for 24 h using the miRCURY cell/urine/CSF kit (Qiagen). Detection of exosome‐specific proteins by Western blot was performed using CD9 and CD63 antibodies (Developmental Studies Hybridoma Bank, University of Iowa). Transwell experiments were performed using SW620 cells as the recipient cells and SW620 *PMIS‐miR‐210* as the donor cells. Six‐well transwell culture vessels were seeded with 2 × 10^5^ recipient cells in the well and 2 × 10^5^, 1 × 10^5^, 0.5 × 10^5^ donor cells in the transwell chamber. After 72 h, cells in the transwell chamber were removed and RNA was isolated from the recipient cells. cDNAs were made and qPCR was performed. Each transwell experiment was repeated three times.

### 2.14 Statistical analysis

For each condition, a minimum of three experiments was performed and error bars were presented as the ±SEM. An independent two‐tailed *t*‐test was used to determine the significance of differences between groups.

### 2.15 Primers used in ChIP and qPCR experiments

#### 2.15.1 ChIP primers

NME1 UTR: 261 bps

F: GCTCTTGGAGCTGTGAGTTCT

R: CCAAGAGTGGAAGGGATGCG

NME1 TSS: 258 bps

F: TAGTCGCGGGAGTGGGTTA

R: CAAGCACTTACAGAGCGCCA

RBCK1 TSS: 197 bps

RBCK1TSS F: GTAGCATTTCCCAGGAGGCA

RBCK1TSS R: GTAGAGGGAGGGCAGGCTAA

RBCK1 UTR: 170 bps

RBCK1UTR F: TGCCAGATCGTGGTACAGAA

RBCK1UTR R: CAGCCCTCCTCTAAGGCAAA

#### 2.15.2 RT‐qPCR primers

RBCK1 fwd5′‐GCA GAT GAA CTG CAA GGA GTA TCA‐3′

RBCK1 rev5′‐TGC AGC ATC ACC TTC AGC AT‐3

## 3 RESULTS

### 3.1 *miR‐210* expression increases in metastatic colorectal cancer

**FIGURE 1:**
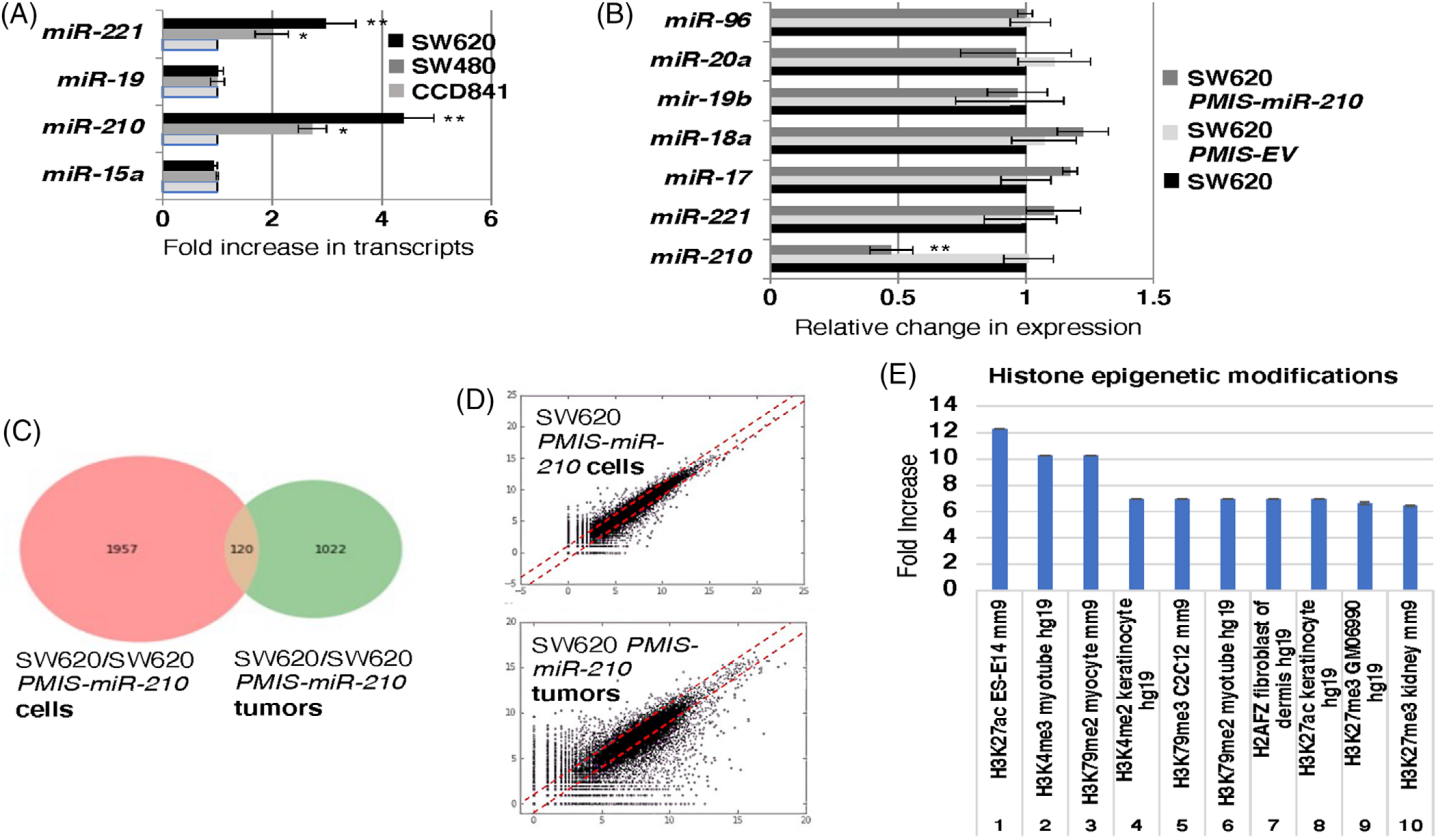
*miR‐210* expression and inhibition profiles in colorectal tumour cells. (A) The metastatic colorectal cancer (CRC) cell line SW620 has the highest level of *miR‐210* transcripts compared to primary CRC SW480 cells (both cell lines originated from the same patient at different stages of cancer progression) and normal colon epithelia (CCD841). (B) *PMIS‐miR‐210* inhibits *miR‐210* expression in SW620 cells, but does not inhibit other miRs. (C and D) RNA sequencing from *PMIS‐miR‐210* SW620 cells revealed different sets of genes regulated by *miR‐210* compared to *PMIS‐miR‐210*‐excised murine xenograft tumours. Only 120 regulated genes were similar in the two sets of RNA‐seq data. (E) These genes were further analyzed for their gene ontology pathways and histone modifying genes including H3K27ac and H3K4me3, which were upregulated. *p < .05; **p < .01

SW480 and SW620 colorectal cancer lines were compared to normal colon epithelium (CCD841) for expression of key molecules and miRs, including *miR‐210*, morphology and growth rates. *miR‐210* expression increases in metastatic tumour cells (SW620) compared to primary tumour cells (SW480) and normal colon epithelia (CC841) (Figure 1A). In addition, DLD1 and Colo320 colorectal tumour cells were also analyzed and showed similar *miR‐210* levels (Figure S1). These data correlate to the survival rate of patients with colorectal cancer, as the survival rate of these patients drops significantly with high levels of *miR‐210* expression.^46^ We focused on SW620 cells for most of the research in this study because these cells have high miR‐210 expression and are associated with more severe colorectal cancer. SW620 cells were transduced with the *PMIS‐miR‐210* inhibitor and a panel of miRs were analyzed for their expression compared to *PMIS‐EV* and non‐transduced cells. *PMIS‐miR‐210* inhibited *miR‐210* in the transduced cells (Figure 1B), accompanied by a slight reduction in growth and mild changes in morphology. However, these changes were not significant and could not be quantitated. DLD1 and Colo320 cells were transduced with *PMIS‐miR‐210* and inhibited *miR‐210* expression (Figure S1).

### 3.2 SW620 cells and tumours present with different gene expression profiles

RNA sequencing of SW620 and SW620‐*PMIS‐miR‐210* cells in culture were compared to SW620 and SW620‐*PMIS‐miR‐210* tumours formed in xenograft mice. The analyses of gene expression revealed only 120 genes were shared between the two groups, highlighting the difference between cells in culture and tumours excised from the mice (Figure 1C,D). We have highlighted histone epigenetic modification pathways affected in both *PMIS‐miR‐210*‐transduced cells and tumours. Gene ontology analyses identified several pathways regulated by miR‐210. Epigenetic modifications were upregulated and these modifications are associated with cancer and microRNAs. We will focus on H3K27ac and H3K4me3, two chromatin modifiers associated with proximal promoters and transcriptional activation in later experiments (Figure 1E).

### 3.3 *PMIS‐miR‐210* inhibits colorectal tumour formation

**FIGURE 2:**
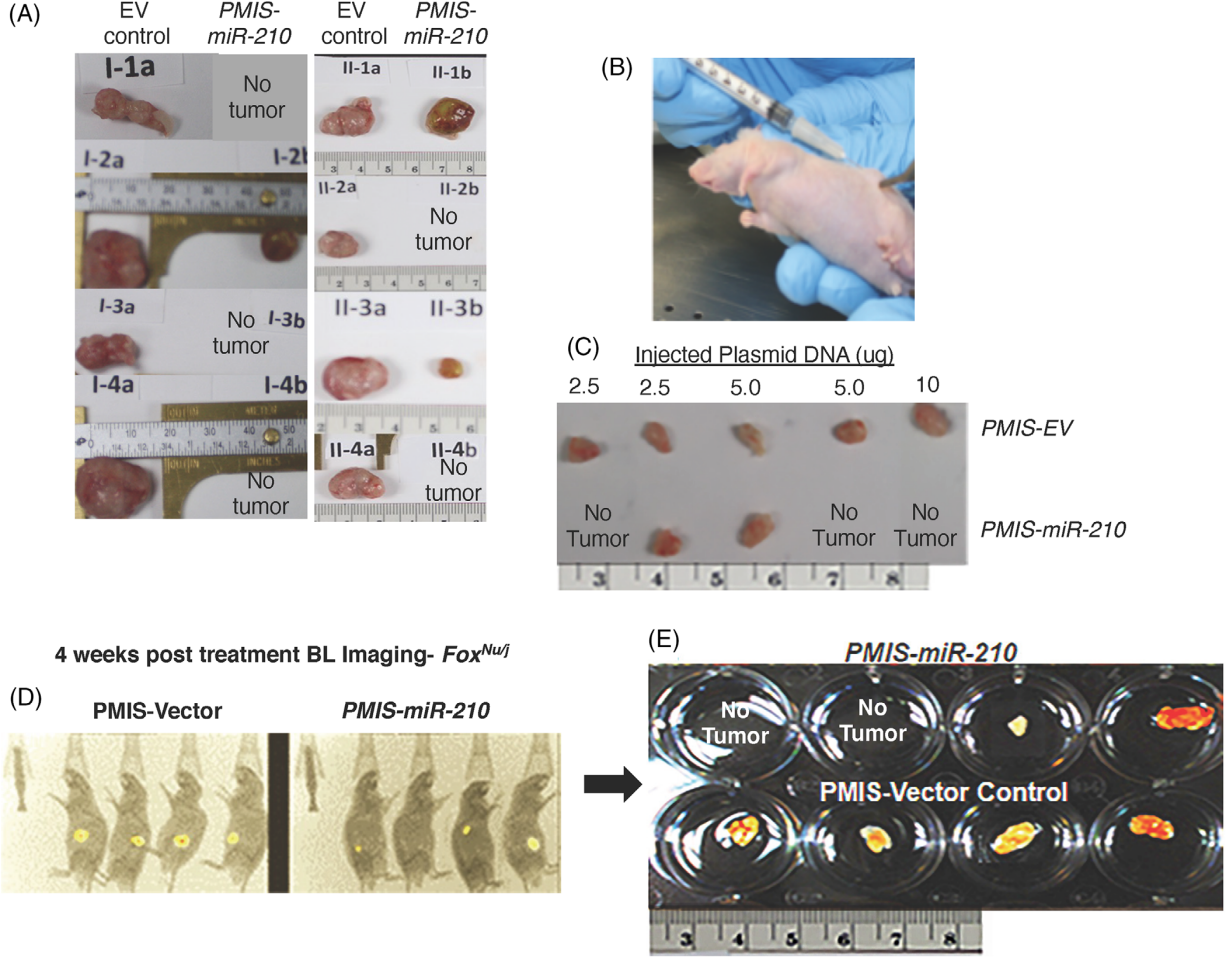
Small colorectal tumours are inhibited or ablated by injection of *PMIS‐miR‐210*. (A) Transduced *PMIS‐miR‐210* or *PMIS‐EV* SW620 cells (5 × 10^5^) were transplanted into the flanks of *Foxn1^nu/j^* nude mice and allowed to grow for 4 weeks, excised and analyzed. Five of the eight *PMIS‐miR‐210* SW620 implanted cells failed to grow tumours compared to controls, which all grew large tumours. (B and C) SW620 cells (5 × 10^5^) were transplanted into nude mice and allowed to grow for 7–10 days and form solid tumours. After 7–10 days, different doses (2.5, 5 or 10 μg) of *PMIS‐miR‐210* or *PMIS‐EV* plasmid DNA were directly injected into the tumour. After 35–40 days, the tumours were removed and analyzed; interestingly, in three of the five mice (at different DNA concentrations) no tumours were found in the *PMIS‐miR‐210*‐injected tumours. (D and E) SW620 cells were transduced with a luciferase construct (5 × 10^5^) to allow for live imaging of tumour formation and after 7–10 days of growth, tumours were injected with 10 μg of *PMIS‐miR‐210* or *PMIS‐EV* plasmid DNA. After 35–40 days, the tumours were imaged, removed and quantitated. In two *PMIS‐miR‐210*‐injected mice, the tumours were not detectable by BLI or visible by dissection.

Because high *miR‐210* expression is associated with colorectal tumours, we first investigated if SW620 cells transduced with *PMIS‐miR‐210* would form tumours in *Foxn1^nu/j^* mice. Lentiviral constructs expressing *PMIS‐miR‐210* or *PMIS‐EV* were used to transduce SW620 cells and the transduced cells were FACS sorted for *PMIS‐miR‐210* and *PMIS‐EV* expression (GFP is expressed from the constructs), which were used to inject into the flanks of *Foxn1^nu/j^* mice. Approximately 5 × 10^5^ cells were placed into each flank and tumours allowed to grow for 4 weeks. Tumours were excised after 4 weeks and measured for growth. Surprisingly, tumours were not formed in five out of eight *PMIS‐miR‐210* SW620 cell transplants and the three tumours that did form were smaller than *PMIS‐EV* controls (Figure 2A).

We next studied if injecting *PMIS‐miR‐210* plasmid DNA directly into the tumour would affect tumour growth. The SW620 tumours (5 × 10^5^ cells) were allowed to form and after 7–10 days of growth, several concentrations of *PMIS‐miR‐210* or *PMIS‐EV* ‘naked’ plasmid DNA were injected into the growing tumour. After 35–40 days of DNA injection, three of the five tumours disappeared using different doses of DNA (Figure 2B,C). In the next set of experiments, SW620 luciferase‐positive cells (5 × 10^5^ cells) were implanted, and tumours were allowed to form and grow for 7–10 days and then injected with PMIS constructs (10 μg) and imaged 35–40 days later to assess tumour growth. Mice with *PMIS‐miR‐210* injections showed reduced bioluminescence compared to controls and in two of four mice with *PMIS‐miR‐210* injections, tumours were not found (Figure 2D,E).

**FIGURE 3:**
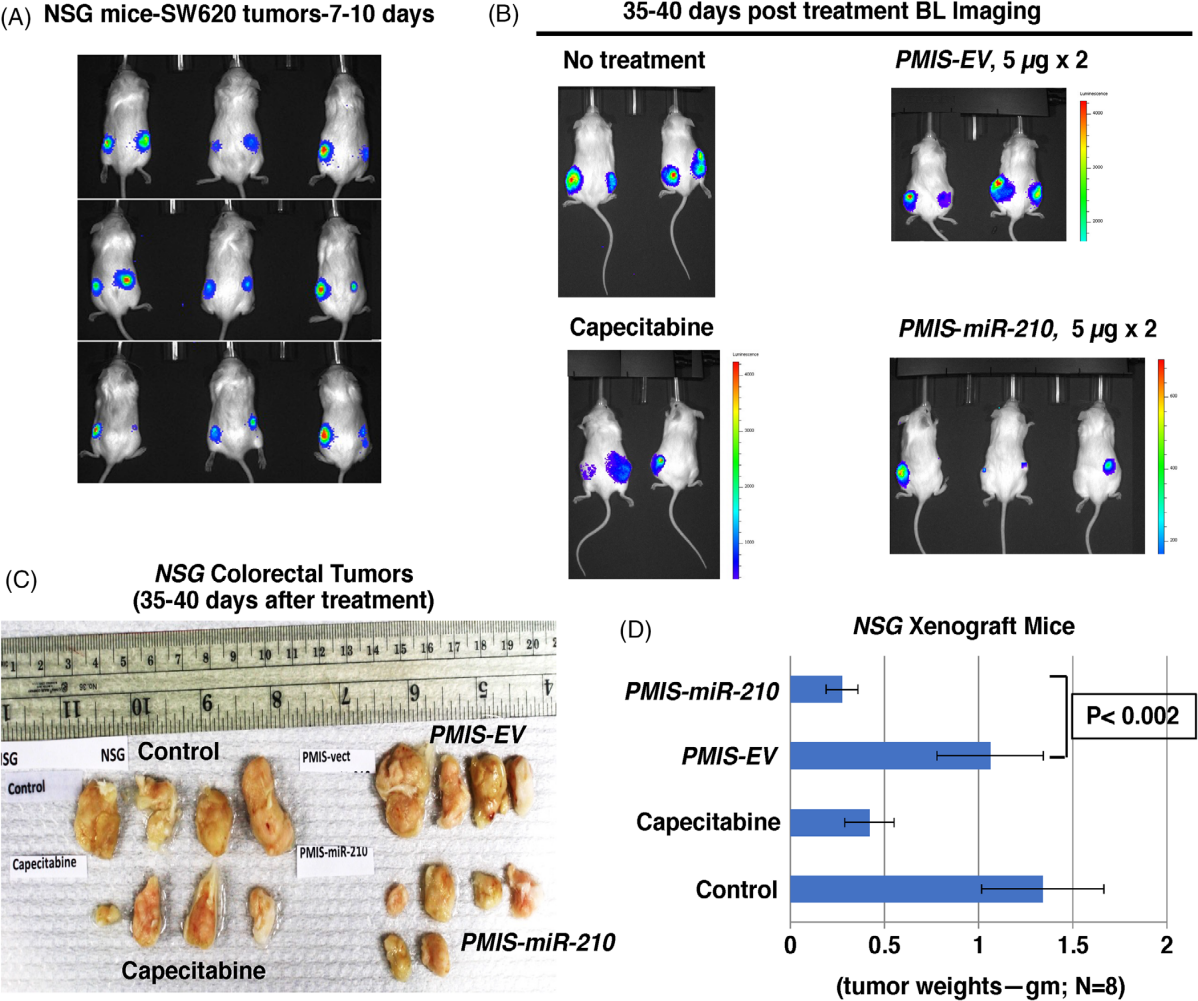
Large colorectal tumours are significantly reduced in *NSG* xenograft mice with *PMIS‐miR‐210* injections. (A) SW620 cells were transduced with a luciferase construct and (1 × 10^6^) cells (2× more than Figure 2) were transplanted into each flank of NSG mice and allowed to grow and imaged after 7–10 days. (B) After 7–10 days, SW620 tumours were injected with 5 μg of *PMIS‐miR‐210* or *PMIS‐EV* plasmid DNA and 5 days later another dose of 5 μg plasmid DNA was administered to the tumours. Capecitabine was given to the mice as a control treatment to compare the efficacy of *PMIS‐miR‐210* treatments. After 35–40 days, the mice were imaged for tumour growth. (C and D) Tumours 35–40 days after treatments were removed and analyzed and tumour weights were recorded, each mouse had two tumours, one on each flank. *N* = 8 per group, except for capecitabine treatment (*N* = 4); Student's *t*‐tests conducted between groups

To further confirm these results, *NSG* mice (immunodeficient, lack T and B cells and NK cells) were transplanted with 1 × 10^6^ SW620 cells (2× more cells than previous experiments) on each flank, and tumours were allowed to grow for 7–10 days. Doubling the number of injected cells resulted in tumours that were almost twice as large as in the previous experiments, and BLI showed the tumours had formed in the mice after 7–10 days (Figure 3A). The mice (randomly selected without bias) were either treated with *PMIS‐EV* or *PMIS‐miR‐210* plasmid DNA with one injection of 5 μg and after 5 days injected again with 5 μg DNA and sacrificed after 35–40 days. These were then compared to a no‐treatment group and a group given capecitabine (Xeloda), which is used to treat patients with metastatic colorectal cancer.^47^ The *NSG* mice treated with *PMIS‐miR‐210* had reduced bioluminescence compared to controls and capecitabine (Figure 3B). The *NSG* mice treated with *PMIS‐miR‐210* showed significantly reduced tumour size and growth compared to controls (Figure 3C,D). Capecitabine treatment caused a decrease in tumour size but was less effective than the *PMIS‐miR‐210* treatment (Figure 3C,D). Tumours were not found in other tissues and the tissues surrounding the tumours were not affected by the treatments.

**FIGURE 4:**
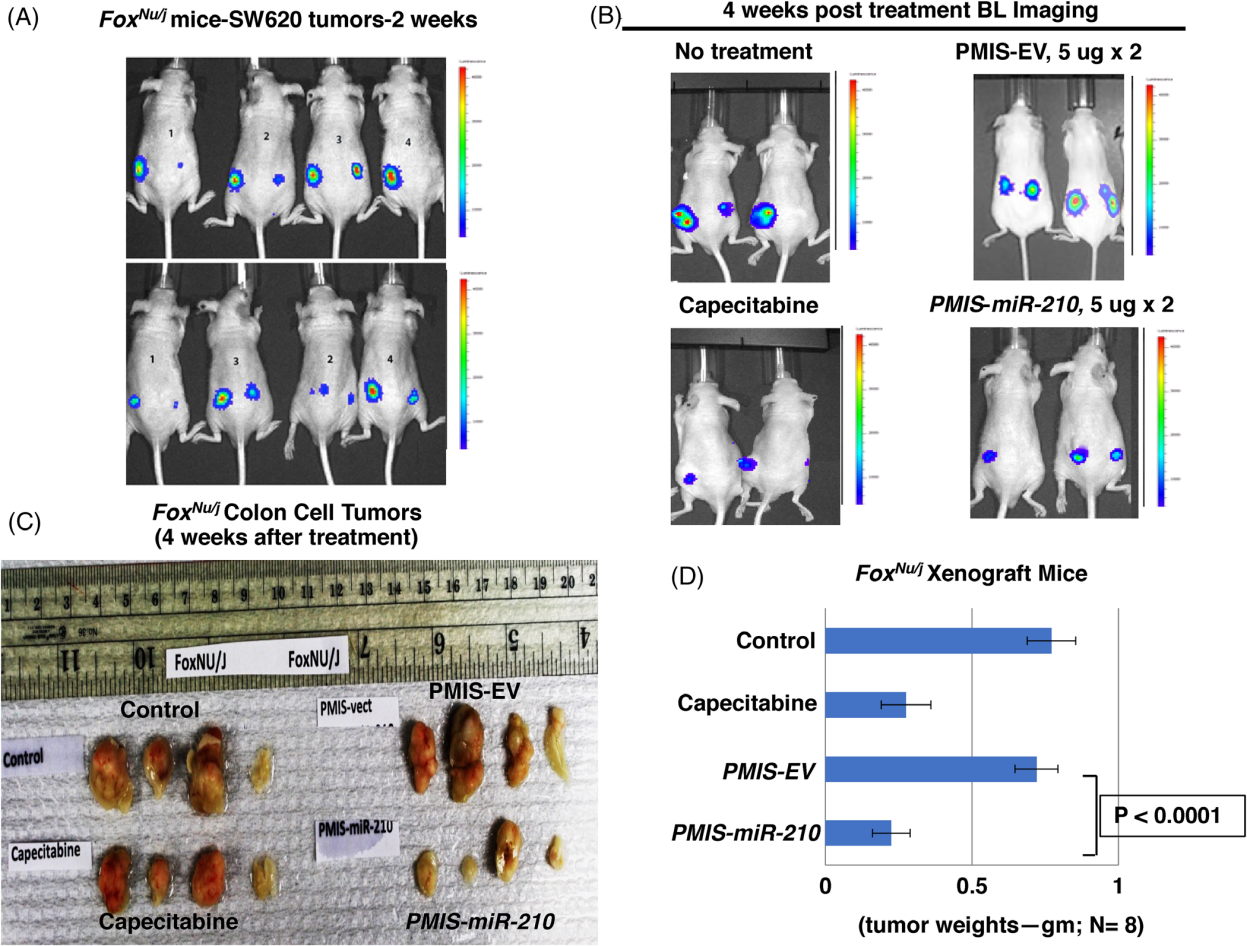
Large colorectal tumours are significantly reduced in *Foxn1^nu/j^* xenograft mice with *PMIS‐miR‐210* injections. (A) SW620 cells were transduced with a luciferase construct and (1 × 10^6^) cells were transplanted into each flank of *Foxn1^nu/j^* mice and allowed to grow and imaged after 7–10 days. (B) After 7–10 days, SW620 tumours were injected with 5 μg of *PMIS‐miR‐210* or *PMIS‐EV* plasmid DNA and 5 days later another dose of 5 μg plasmid DNA was administered to the tumours. Capecitabine was given to the mice as a control treatment to compare the efficacy of *PMIS‐miR‐210* treatments. After 35–40 days, the mice were imaged for tumour growth. (C and D) Tumours 35–40 days after treatments were removed and analyzed and tumour weights were recorded, each mouse had two tumours, one on each flank. *N* = 8 per group; *N* = 4 for capecitabine treatment; Student's *t*‐tests conducted between groups

These experiments were repeated using *Foxn1^nu/j^* mice (immunodeficient, lack T cells) and the same parameters used for the *NSG* mice. The growing tumours were visualized in the mice after 7–10 days (Figure 4A). As with *NSG* mice, these mice were treated under the same protocol. The mice treated with *PMIS‐miR‐210* and capecitabine both showed reduced tumour bioluminescence (Figure 4B). The tumours were removed after 35–40 days, measured, weighed, and analyzed. *Foxn1^nu/j^* mice treated with *PMIS‐miR‐210* had significantly reduced tumour size and growth compared to controls (Figure 4C,D). Capecitabine treatment showed a decrease in tumour size but was less effective than the *PMIS‐miR‐210* treatment (Figure 4C,D). These data demonstrate the efficacy of the *PMIS‐miR‐210* treatment for growing colorectal tumours in two different immunocompromised mice models. Interestingly, treatment of smaller tumours (5 × 10^5^ vs. 1 × 10^6^ cells) resulted in several *PMIS‐miR‐210* tumours completely disappearing, demonstrating that treatments of smaller tumours have a better outcome. However, in this report tumours were harvested after only 35–40 days post treatment, and a better response may be achieved after longer treatment times. Tumour growth was measured using a caliper and recorded prior to injection and 1 week after injection (Figure S2). Furthermore, toxicity screening for ALT and AST in the blood and liver of these mice showed no elevated levels of these markers, suggesting that longer treatment times with *PMIS‐miR‐210* would be safe (Figure S3).

### 3.4 *PMIS‐miR‐210* is widely expressed in the injected colorectal tumours

**FIGURE 5:**
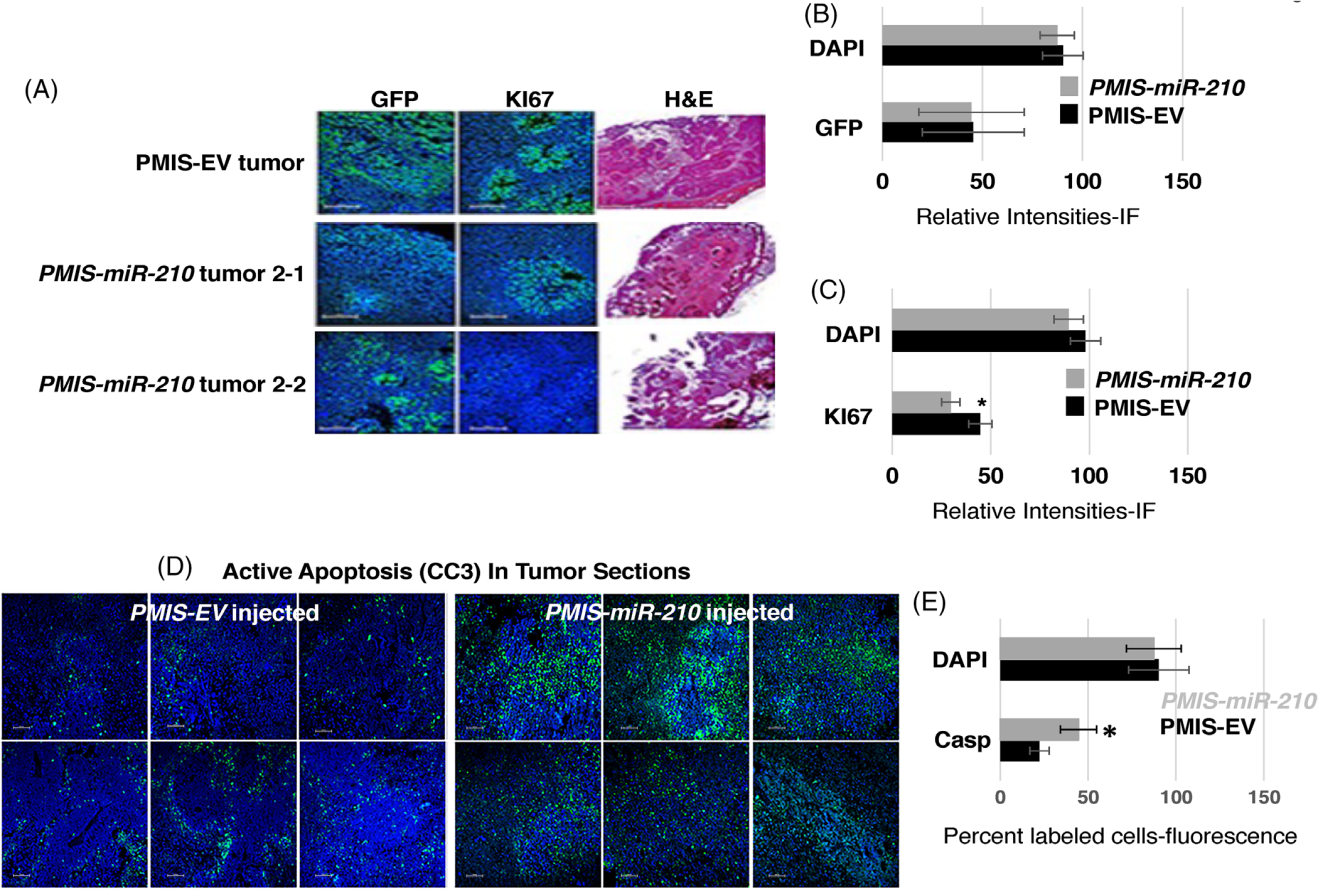
*PMIS‐miR‐210* is widely expressed in the injected tumours. (A) *PMIS‐EV* and *PMIS‐miR‐210* injected SW620 tumours from *Foxn1^nu/j^* (from Figure 4) mice were sectioned and stained for GFP, and Ki67 expression. GFP is expressed from both *PMIS‐EV* and *PMIS‐miR‐210* expression constructs. (B and C) The relative intensities of GFP and Ki67 immunofluorescence were calculated using ImageJ and values shown in the graphs. Four independent sections for each of the two tumours per group were used, *N* = 4 each. (D) Serial sections from the tumours were probed for cleaved caspase 3 (CC3) expression to demonstrate active apoptosis. (E) CC3 expression measured by immunofluorescence was significantly increased in SW620 tumours expressing *PMIS‐miR‐210*. *N* = 3; *p < .05

To determine if the tumour cells were expressing the *miR‐210* inhibitor, we assayed for GFP expression as both *PMIS‐EV* and *PMIS‐miR‐210* expression constructs also express *GFP*. GFP was expressed throughout the tumours in both the *PMIS‐EV* and *PMIS‐miR‐210* treatment groups (Figure 5A,B). Multiple tumours and sections were analyzed for GFP expression and representative GFP staining is shown in Figure 5A. The expression of Ki67, a marker for proliferating cells, was decreased in the *PMIS‐miR‐210* tumours compared to *PMIS‐EV* tumours (Figure 5A,C). Several tumours were stained with H&E to show cellular and tissue structures. The *PMIS‐miR‐210*‐treated tumours showed a less dense tissue with decreased vascularization and structure (Figure 5A). These data demonstrate that direct injection of *PMIS‐miR‐210* plasmid DNA into cancer tissue results in widespread *PMIS‐miR‐210* expression. *miR‐210* expression was inhibited in the tumours by *PMIS‐miR‐210* (2.7‐fold decrease compared to EV control).

### 3.5 Inhibition of *miR‐210* activates the apoptotic pathway in colorectal tumours

The colorectal tumours in all xenograft mice were either reduced in size or disappeared after *PMIS‐miR‐210* treatment, while cell proliferation was moderately reduced compared to control groups. Therefore, we assayed tumour sections for active apoptosis using cleaved caspase 3 (CC3) expression. In *PMIS‐EV*‐injected sections (six serial sections), only a small amount of CC3 was expressed; however, *PMIS‐miR‐210*‐injected sections contained high levels of CC3 expression, indicating active apoptosis in these tumours (Figure 5D). Apoptosis was quantified by RNA sequencing (*Casp3* increased two‐fold in *PMIS‐miR‐210* tumours, compared to controls) and qPCR (*Casp3* increased 2.7‐fold in *PMIS‐miR‐210* tumours, compared to controls). Furthermore, fluorescence intensity was measured in the sections and showed a significant increase in CC3 expression in the *PMIS‐miR‐210* tumours compared to controls (Figure 5E). Clearly, apoptosis can account for the decrease in tumour growth; however, there are other mechanisms and targets of *miR‐210* that play a role in tumour growth.

### 3.6 *miR‐210* inhibits *NME1* expression in colorectal tumours

**FIGURE 6:**
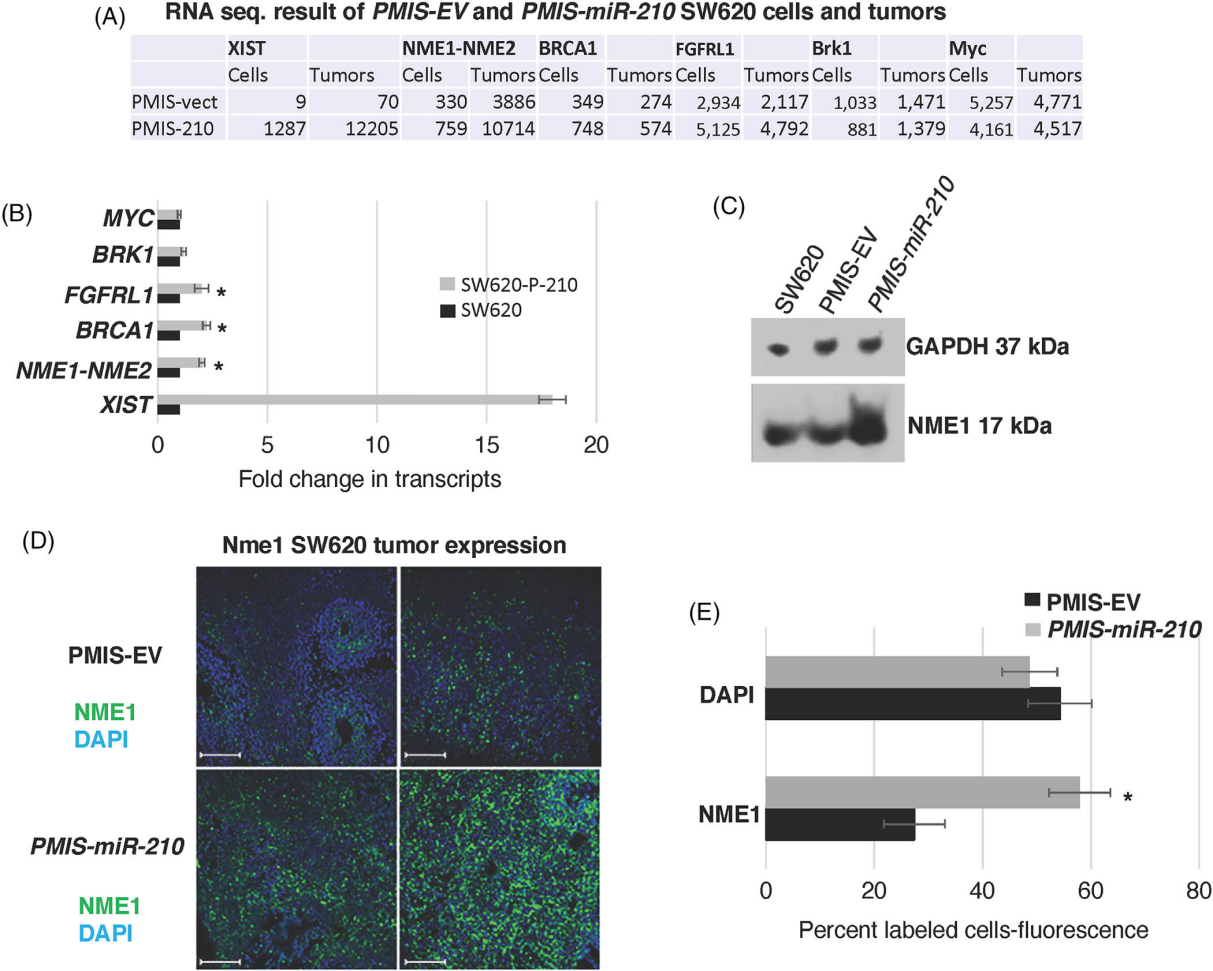
The tumour suppressor *NME1* is regulated by *miR‐210*. (A) RNA‐seq from SW620 cells and tumours were specifically analyzed for *XIST*, *NME1*, *BRCA1*, *FGFRL1*, *BRK1* and *MYC* expression with and without *PMIS‐miR‐210* expression. (B) Transcript levels of the genes in panel A were validated by qPCR in SW620 cells with and without *PMIS‐miR‐210* expression. *N* = 3. (C) NME1 protein levels were increased in *PMIS‐miR‐210*‐transduced SW620 cells compared to *PMIS‐EV* and untreated cell controls. (D and E) NME1 expression measured by immunofluorescence was significantly increased in SW620 tumours expressing *PMIS‐miR‐210*. *N* = 6; *p < .05

RNA sequencing of SW620 cells and tumours transduced with *PMIS‐EV* or *PMIS‐miR‐210* revealed an increase in *NME1* transcripts in the *PMIS‐miR‐210* cells and tumours compared to controls (Figure 6A). These results were validated by qPCR from RNA isolated from the tumours and by an increase in protein expression (Figure 6B,C). *NME1* was significantly increased in *PMIS‐miR‐210* tumour sections compared to *PMIS‐EV* controls (Figure 6D,E). NME1 can regulate gene expression by binding to promoter/enhancer regions of genes that contribute to tumour metastasis suppressor function.^42^ *miR‐210* appears to repress *NME1* expression as a partial mechanism to regulate tumorigenicity. These results were also observed in DLD1 and Colo320 cells (Figure S1). However, *miR‐210* does not bind to the *3′UTR* of *NME1*, and we determined that the regulation of *NME1* occurs through epigenetic modification of the genome.

### 3.7 *miR‐210* regulates the amount of *XIST* transcripts in cells

**FIGURE 7:**
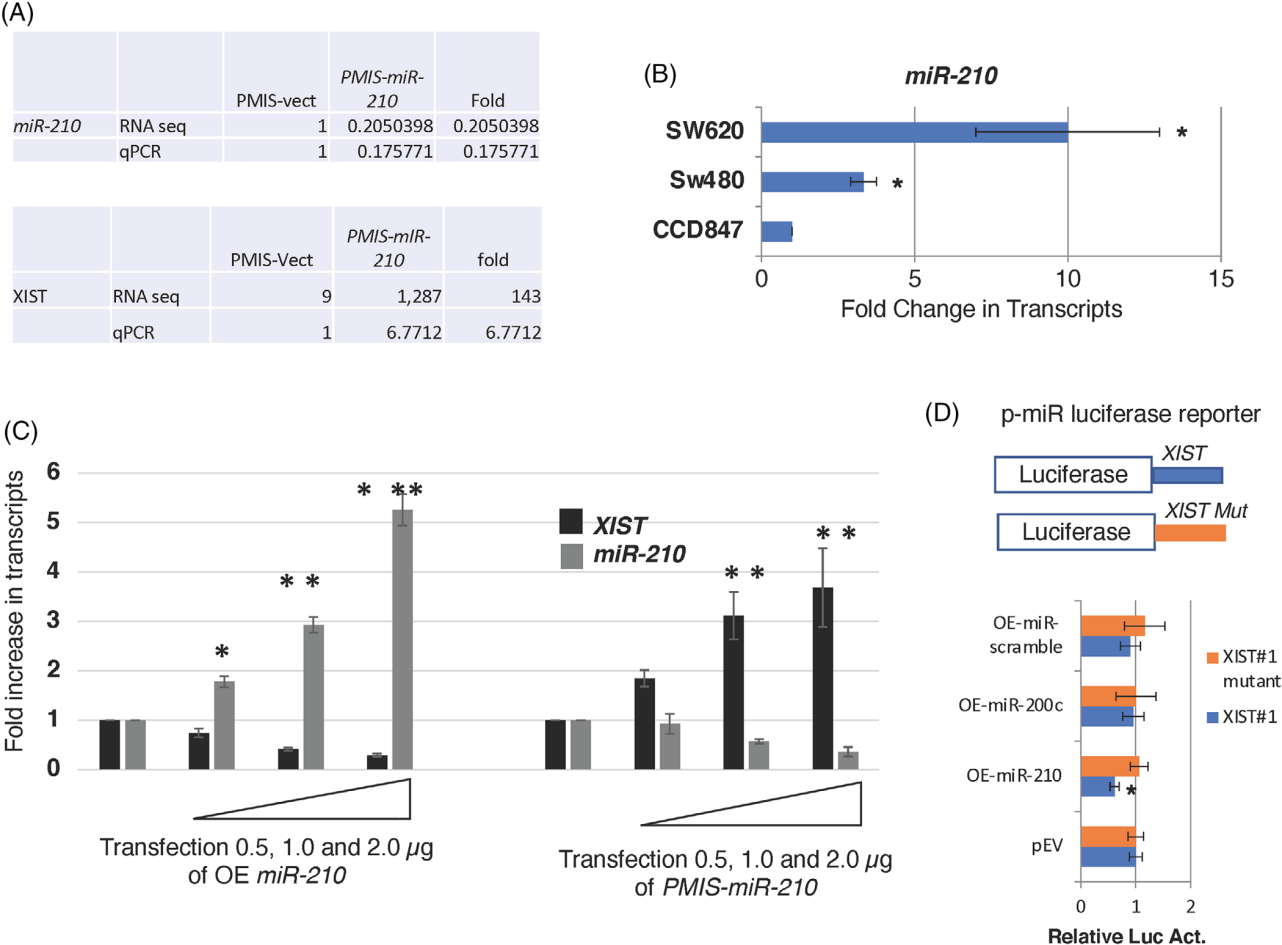
*miR‐210* regulates *XIST* transcript levels. (A) RNA‐seq and qPCR showing that *miR‐210* levels were inhibited over 80% in SW620 cells and *XIST* transcript were increased 143‐fold in *PMIS‐miR‐210*‐transduced SW620 cells. (B) *miR‐210* expression increases in metastatic colorectal SW620 cells. (C) Demonstration of an inverse relationship between *miR‐210* and *XIST* expression. Increasing amounts of transfected *miR‐210* expression vector resulted in decreasing amounts of endogenous *XIST* transcripts. Increasing amounts of transfected *PMIS‐miR‐210* correlate with decreased *miR‐210* and increased *XIST* transcripts. The mouse (Mu) and Human (Hu) *XIST* transcripts contain *miR‐210* binding sites. (D) Dual luciferase reporter assays in 293 cells with a *Luc‐XIST* reporter, 2.5 μg (contains *miR‐210* binding sequence) and *Luc‐XIST mut* reporter 2.5 μg (*miR‐210* binding sequences mutated). *miR‐210*, *miR‐200c*, scrambled and empty vector (EV) expression constructs (5 μg) were transfected in 293 cells. Luciferase was measured after 48 h post transfection. *N* = 3, *p < .05; **p < .01

We show a direct correlation of *miR‐210* expression and the levels of the long non‐coding RNA (LncRNA X‐inactive specific transcript) *XIST* transcripts. Both RNA‐seq and qPCR of RNA isolated from tumours and transduced SW620 cells demonstrate that *PMIS‐miR‐210* inhibits *miR‐210* by over 80%, which results in an increase in *XIST* transcripts (Figure 7A). As mentioned previously, increasing *miR‐210* transcripts correlates with advanced stages of colorectal cancer and *miR‐210* is highly expressed in the SW620 metastatic colon cancer cell line compared to the non‐metastatic SW480 colon cancer cell line and normal colon epithelium (Figure 7B). To further demonstrate the inverse correlation of *miR‐210* and *XIST*, 293 cells were transfected with increasing concentrations of plasmid DNA expressing *miR‐210* (overexpressing [OE] *miR‐210*), and endogenous *XIST* was decreased in a dose‐dependent response (Figure 7C). When endogenous *miR‐210* was decreased with increasing amounts of plasmid DNA expressing *PMIS‐miR‐210*, endogenous *XIST* was increased in a dose‐dependent response (Figure 7C). Both murine and human *XIST* contain multiple *miR‐210* binding elements, indicating that *miR‐210* binds to *XIST* to regulate *XIST* transcript levels (Figure 7C). A dual luciferase reporter construct containing *XIST* sequences (with *miR‐210* binding elements) and *XIST* sequences with the *miR‐210* binding elements mutated (Mut) downstream of the luciferase gene were used to assay for *miR‐210* activity regulating *XIST*. OE of *miR‐210* inhibited luciferase activity from the *Luc‐XIST* reporter, but not from the *Luc‐XIST* mut (Figure 7D). As controls, OE of *miR‐200c*, EV or miR‐scrambled expression construct had no effect on luciferase activity (Figure 7D). A previous report suggested that *miR‐210* bind to *XIST* and regulate *XIST* transcript levels.^48^ Our results show a direct regulation of *XIST* by *miR‐210*.

### 3.8 *miR‐210* controls the epigenetic regulation of *NME1* through *XIST*

**FIGURE 8:**
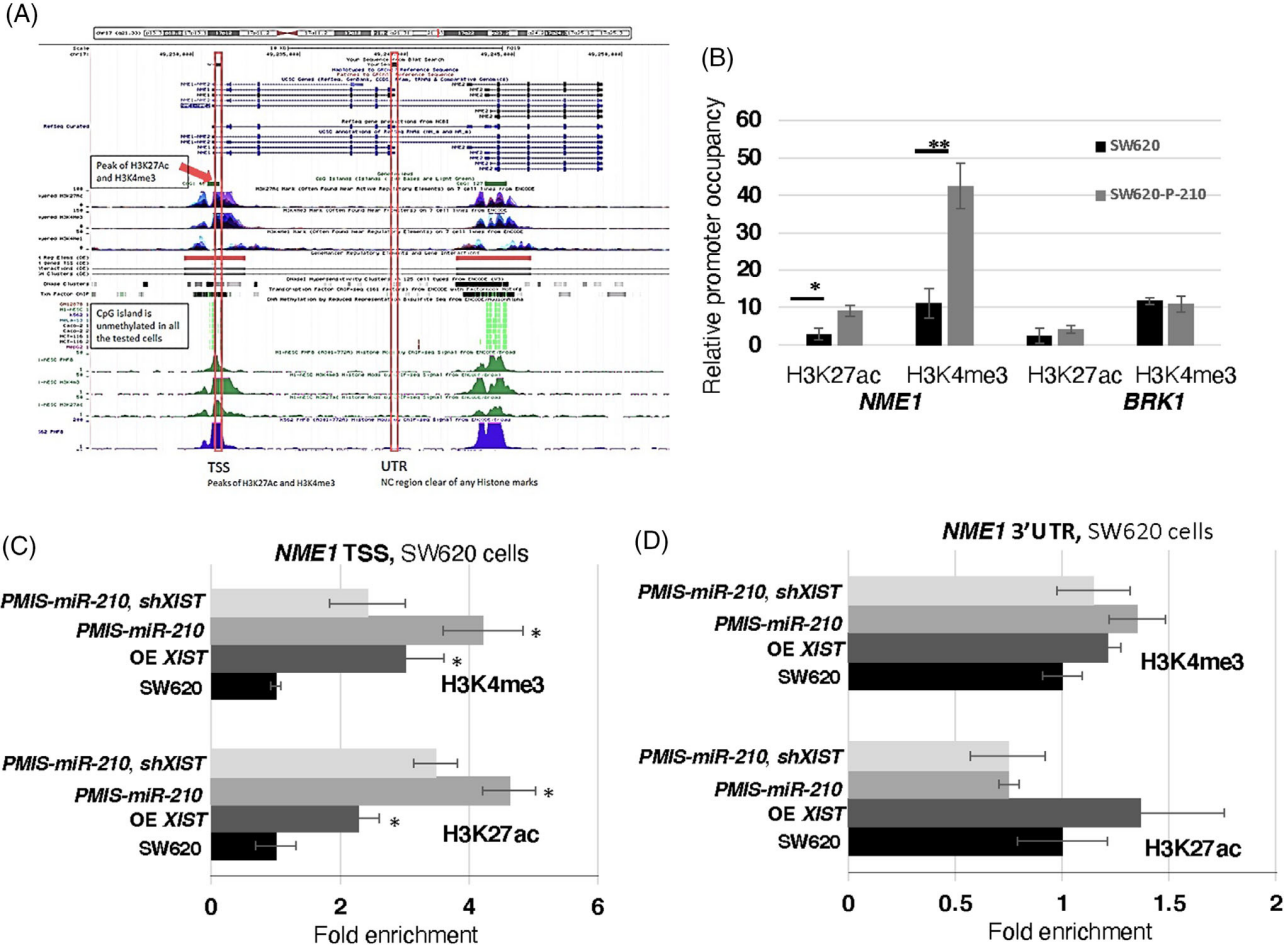
Epigenetic regulation of *NME1* by *miR‐210* and *XIST*. (A) Analyses of epigenetic regulators activating *NME1* expression revealed that H3K27Ac and H3K4me3 both marked the *NME1* transcription start site (TSS) and identified *NME1* as an actively transcribed gene. (B) ChIP‐qPCR experiments demonstrate in SW620 cells transduced with *PMIS‐miR‐210* that both H3K27Ac and H3K4me3 marks were increased at the proximal promoter. As a control, Brk1 was not affected, *N* = 4. (C and D) ChIP‐qPCR experiments in SW620 cells demonstrate that *XIST* overexpression increased H3K27ac and H3K4me3 chromatin marks at the *NME1* TSS but not at the *NME1* 3′UTR, used as a control, *N* = 5 (D). SW620 cells transfected with *PMIS‐miR‐210* increased both epigenetic marks at the *NME1* TSS (C) but not at the *NME1* 3′UTR (D). SW620 cells co‐transfected with *PMIS‐miR‐210* and *shXIST*, decreased the epigenetic marks compared to *PMIS‐miR‐210* alone, indicating that *miR‐210* inhibition of *XIST* plays a role in *NME1* epigenetic regulation. *N* = 5, *p < .05; **p < .01

We wanted to check if the *NME1* proximal promoter was activated by *miR‐210* inhibition using two epigenetic histone markers, H3K4me3 and H3K27ac. ChIP‐sequencing previously identified H3K27ac and H3K4me3 peaks upstream of the *NME1* transcription start site (TSS) (Figure 8A; ENCODE Consortium). We used ChIP‐qPCR to determine if these epigenetic markers were increased after *miR‐210* inhibition in SW620 cells. Both H3K27ac and H3K4me3 were enriched at *NME1* promoter elements in *PMIS‐miR‐210* SW620 cells (Figure 8B). As a control, the *BRK1* gene was not affected. These epigenetic regulators are associated with active sites of transcription^49^ and increased transcription elongation and enhancer activity at tumour suppressor genes.^50^

*XIST* was increased over 150‐fold in RNA‐seq experiments and over 15‐fold in qPCR assays of RNA isolated from *PMIS‐miR‐210* cells and tumours (Figure 8A,B). While *XIST* RNA is required for X chromosome inactivation (XCI), it can also suppress cancer in vivo.^51^ Because *XIST* expression was significantly increased in SW620 cells and tumours expressing *PMIS‐miR‐210*, we asked if *XIST* also contributed to epigenetic regulation of *NME1*. ChIP‐qPCR experiments were performed for both H3K3me3 and H3K27ac in SW620 cells expressing *PMIS‐miR‐210*, OE of *XIST* and *PMIS‐miR‐210* combined with *XIST* shRNA (to knockdown *XIST* transcripts). Overexpression of *XIST* resulted in two to three‐fold enrichment of H3K27ac and H3K4me3 at the *NME1* proximal promoter, demonstrating a direct effect on histone modifications by *XIST* (Figure 8C). *PMIS‐miR‐210* further increased the requirement of these epigenetic factors at the *NME1* promoter associated with an increase in *XIST* expression (Figure 8C). To validate the role of *XIST*, *XIST* expression was reduced using an shRNA to *XIST* and in combination with *PMIS‐miR‐210* demonstrated a decrease in the enrichment of these epigenetic factors at the *NME1* promoter, compared to *PMIS‐miR‐210* alone or *XIST* OE (Figure 8C). As controls, we also determined if these factors were enriched at the *NME1* 3′UTR and found that they were not associated with this part of the gene (Figure 8D). These data suggest that *miR‐210* inhibition increases *XIST* expression, which can facilitate H3K4me3 and H3K27ac deposition at the *NME1* promoter, activating transcription of *NME1*. Thus, we propose that *miR‐210* regulates the degradation of *XIST* in cells as a mechanism to promote colorectal cancer and reducing *NME1* expression.

### 3.9 *PMIS‐miR‐210* inhibitor is transmitted from transfected tumour cells to other tumour cells by extracellular vesicles

**FIGURE 9:**
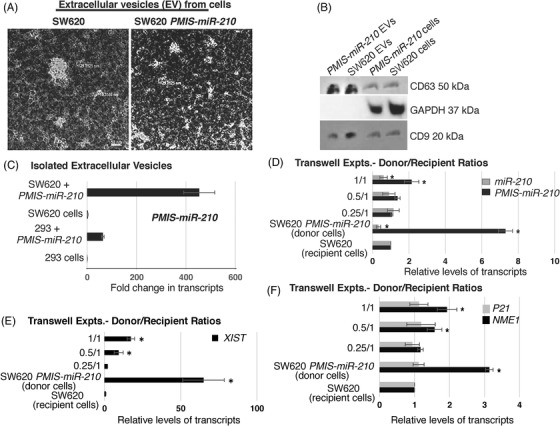
Cell‐to‐cell transfer of *PMIS‐miR‐210* by extracellular vesicles (ECVs). (A) Transmission electron microscopy (TEM) photo of ECVs secreted from SW620 and SW620 *PMIS‐miR‐210* cells. (B) Isolated ECVs were probed for CD63 and CD9 to prove their identity. (C) ECVs isolated from *PMIS‐miR‐210*‐transduced SW620 and 293 cells were probed by qPCR for *PMIS‐miR‐210* transcripts. SW620 *PMIS‐miR‐210* cells secreted high levels of *PMIS‐miR‐210* in ECVs compared to controls and higher levels than 293 *PMIS‐miR‐210* cells. (D) Transwell experiments demonstrate that SW620 cells transduced with *PMIS‐miR‐210* (donor cells) could transfect and express *PMIS‐miR‐210* in SW620 recipient cells. A ratio of 1/1 (2 × 10^5^ donor cells to 2 × 10^5^ recipient cells) showed that the recipient cells expressed the *PMIS‐miR‐210* transcript after 72 h. Furthermore, *miR‐210* expression was decreased in the recipient cells at a 1/1 ratio. The concentration of donor cells was decreased by half at 1 × 10^5^ (0.5/1), while the recipient cell concentration was maintained at 2 × 10^5^ and the recipient cells showed less expression of *PMIS‐miR‐210* compared to the 1/1 ratio. *miR‐210* expression was decreased but less than the 1/1 ratio. A ratio of 0.25 donor/1 recipient cell did not show a significant effect. As controls, the donor cells express high levels of *PMIS‐miR‐210* and decreased levels of *miR‐210*, while the recipient cells alone did not express *PMIS‐miR‐210*. (E) Identical experiments show that the long non‐coding RNA (LncRNA X‐inactive specific transcript) *XIST* levels were increased in the recipient cells at 1/1 and 0.5/1 ratios. *XIST* is highly expressed in the SW620 *PMIS‐miR‐210*‐transduced cells but not in the SW620 cells. (F) The tumour suppressor *NME1* is also increased in the recipient cells at 1/1 and 0.5/1 ratios. As a control, we show that *P21* was not affected as it is not a target of *miR‐210*. *NME1* transcripts are increased in the SW620 *PMIS‐miR‐210*‐transduced donor cells. *N* = 3 for transwell experiments; *p < .05; **p < .01

The levels of GFP staining suggested that most tumour cells were expressing *PMIS‐miR‐210*. Because of the high level of *PMIS‐miR‐210* expression in tumours, we asked if the transfected tumour cells were packaging the *PMIS‐miR‐210* transcripts into extracellular vesicles (ECVs), which could then be transfecting other cells within the tumours. To determine if SW620 cells actively packaged *PMIS‐miR‐210* into ECVs, ECVs were isolated from SW620 cells transduced with *PMIS‐miR‐210* (integrated *PMIS‐miR‐210* into the chromatin, not plasmid DNA transfection) and visualized by TEM (Figure 9A). ECVs were found secreted by SW620 cells. Isolated ECVs were identified by their expression of CD63 and CD9 (Figure 9B). ECVs isolated from 293 and SW620 cells transduced with *PMIS‐miR‐210* were analyzed for *PMIS‐miR‐210* transcripts, and both transduced cell lines produced high levels of *PMIS‐miR‐210* containing ECVs (Figure 9C). In initial experiments, the isolated ECVs from *PMIS‐miR‐210*‐transduced 293 and SW620 cells were used to determine if *miR‐210* levels were affected in SW620 cells after incubation with the ECVs. ECVs from *PMIS‐miR‐210‐*transduced 293 cells were able to inhibit *miR‐210* levels in SW620 cells at two different concentrations (Figure S4A). Furthermore, ECVs isolated from *PMIS‐miR‐210‐*transduced SW620 cells were able to inhibit endogenous *miR‐210* in untreated SW620 cells (Figure S4A). We next asked if *PMIS‐miR‐210* from ECVs would increase *NME1* transcript levels, because we identified *NME1* as a potential target of *miR‐210* in RNA‐seq experiments. ECVs from both *PMIS‐miR‐210*‐transduced 293 and SW620 cells incubated with untreated SW620 cells increased *NME1* transcript levels (Figure S4B). As a control, *BRK1* transcript levels were not changed, as it is not a target of *miR‐210* (Figure S4B). Furthermore, *PMIS‐miR‐210* ECVs from these transduced cells increased the levels of *FGFRL1* and *XIST* transcripts (Figure S4C).

We used transwell experiments to determine if ECVs produced by the SW620 cells transduced with *PMIS‐miR‐210* (donor cells) could transfect and express *PMIS‐miR‐210* in SW620 recipient cells. A ratio of 1/1 (2 × 10^5^ donor cells to 2 × 10^5^ recipient cells) showed that the recipient cells expressed the *PMIS‐miR‐210* transcript after 72 h (Figure 9D). Furthermore, *miR‐210* expression was decreased in the recipient cells at a 1/1 ratio (Figure 9D). The concentration of donor cells was decreased by half (0.5/1 or 1 × 10^5^ donor cells/2 × 10^5^ recipient cells, and as expected the recipient cells showed less expression of *PMIS‐miR‐210* compared to the 1/1 ratio (Figure 9D). *miR‐210* expression was decreased but less than the 1/1 ratio. A ratio of 0.25/1 cells did not show a significant effect. As controls, the donor cells express high levels of *PMIS‐miR‐210* and decreased levels of *miR‐210*, while the recipient cells alone did not express *PMIS‐miR‐210* (Figure 9D). Identical experiments were performed and *XIST* levels were increased in the recipient cells at 1/1 and 0.5/1 ratios (Figure 9E). *XIST* is highly expressed in the SW620 *PMIS‐miR‐210* transduced cells, but not in the SW620 cells (Figure 9E). In identical experiments, we show that the tumour suppressor *NME1* is also increased in the recipient cells at 1/1 and 0.5/1 ratios (Figure 9F). As a control, we show that P21 was not affected as it is not a target of *miR‐210*. *NME1* transcripts were increased in the SW620 *PMIS‐miR‐210*‐transduced donor cells (Figure 9F).

In addition, donor cells were treated with EV inhibitor neticonazole for 48 h and then transwell experiments were performed to demonstrate that *PMIS‐miR‐210* was transferred to recipient cells via ECVs. Experiments were performed as in Figure 9. Recipient cells in the transwell experiments did not express *PMIS‐miR‐210*, and *miR‐210* levels were not affected in the recipient SW620 cells after donor cell treatments (Figure S5A). As a control, the treated SW620 *PMIS‐miR‐210* donor cells retained expression of *PMIS‐miR‐210* and inhibited *miR‐210* (Figure S5A). We also measured the level of *XIST* after donor cell treatments in the recipient cells and found *XIST* levels were not increased; however, *XIST* levels remained high in the SW620 *PMIS‐miR‐210* donor cells after treatment (Figure S5B). These data suggest that extracellular vesicle transfer of *PMIS‐miR‐210* contributes to increased tumour expression of *PMIS‐miR‐210*.

### 3.10 The cellular location and levels of *XIST* are controlled by *miR‐210*

**FIGURE 10:**
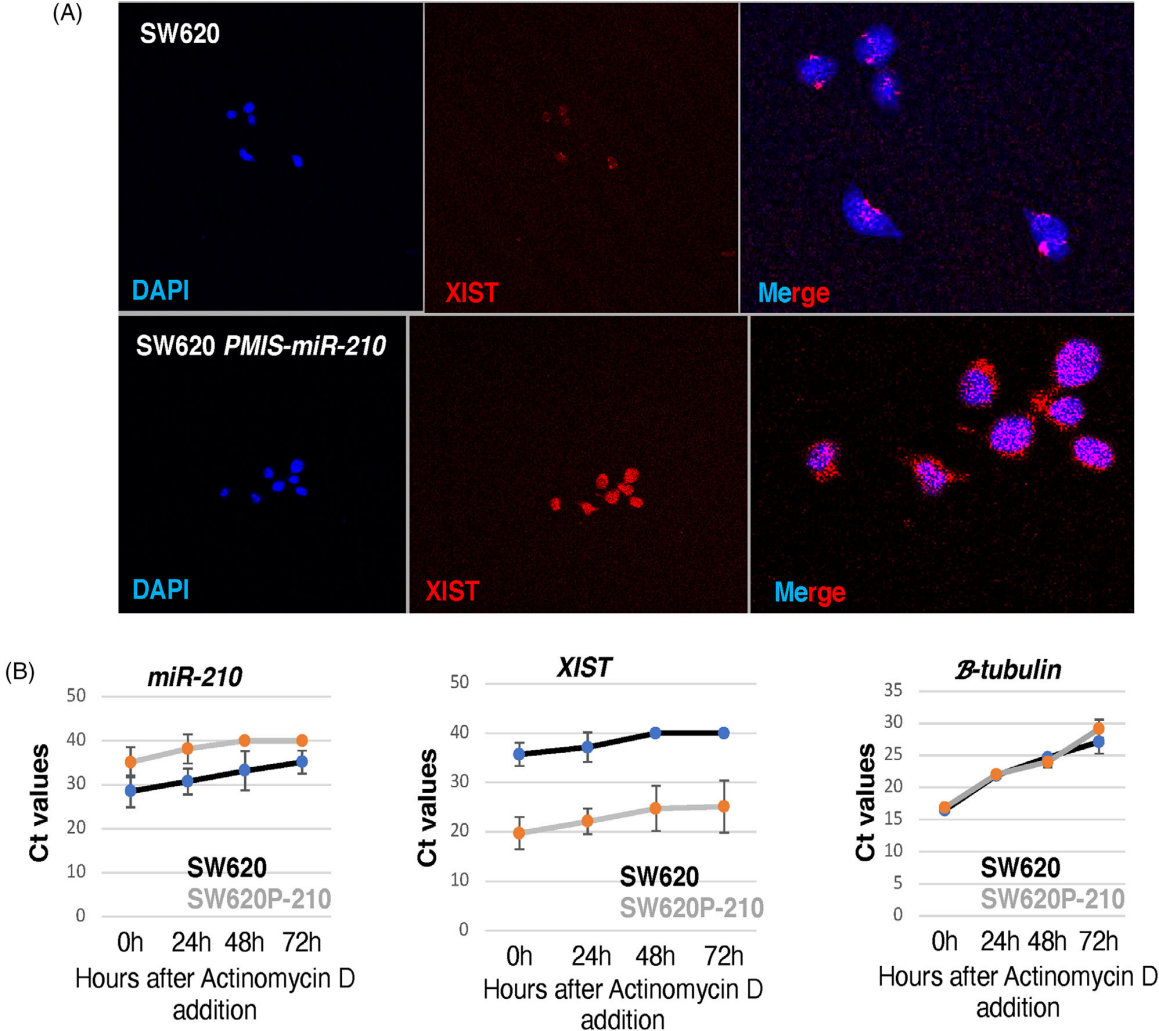
*miR‐210* controls cytoplasmic and nuclear accumulation of *XIST*. (A) Immunofluorescence of *XIST* transcripts in SW620 cells and *PMIS‐miR‐210*‐transduced SW620 cells by in situ hybridization. The nuclei are stained with DAPI (blue). (B) *miR‐210* and *XIST* transcripts are decreased in SW620 cells after treatment with actinomycin D, measured by qPCR and *C*_t_ values plotted. However, *XIST* transcripts are relatively stable in *PMIS‐miR‐210*‐transduced SW620 cells, while *miR‐210* transcripts are rapidly degraded in the presence of actinomycin D. β‐Tubulin was used as a control and rapidly degraded after 3 days of actinomycin treatment. *N* = 3; representative experiment shown

It is well known that *XIST* transcripts are expressed in the nucleus and several reports suggest that it is strictly localized to the nucleus and not found in the cytoplasm.^52^ In addition, a recent report suggests that U1 snRNP regulates chromatin retention of non‐coding RNAs except for *XIST*.^53^ Therefore, it was unknown how miRs in the cytoplasm could interact with *XIST* or if *XIST* was exported to the cytoplasm. Our data suggest that *XIST* transcripts bind *miR‐210* and are rapidly degraded; therefore, we asked if inhibiting *miR‐210* resulted in *XIST* transcripts in the cytoplasm and a subsequent increase in nuclear *XIST*. In SW620 cells containing high levels of *miR‐210, XIST* transcripts were observed confined to the nucleus (Figure 10A). However, SW620 cells expressing *PMIS‐miR‐210* showed an increase in both nuclear and cytoplasmic *XIST* transcripts (Figure 10A). These data demonstrate for the first time that *miR‐210* controls the cytoplasmic levels of *XIST* and that *XIST* transcripts can be found in the cytoplasm when *miR‐210* is inhibited by *PMIS‐miR‐210*.

We next asked if inhibiting transcription using actinomycin D would influence the levels of *miR‐210* and *XIST* in SW620 cells. After 3 days of actinomycin D treatment, *miR‐210* levels were decreased in SW620 cells; however, detectable levels of *miR‐210* remain (Figure 10B). Inhibition of *miR‐210* by *PMIS‐miR‐210* was efficient and after 2 days of actinomycin D treatment, no detectable levels of *miR‐210* were observed in the cells (Figure 10B). Conversely, *XIST* levels were low in SW620 cells and after 2 days of actinomycin D treatment, *XIST* transcripts were not detected (Figure 10B). However, in cells expressing *PMIS‐miR‐210*, the levels of *XIST* remain high even after 3 days of actinomycin D treatments (Figure 10B). Thus, *XIST* degradation depends on *miR‐210*, and the regulation of *XIST* levels is tightly controlled by *miR‐210* in SW620 cells. Thus, high levels of *miR‐210* expression in SW620 cells reduce *XIST* transcripts and inhibit *NME1* expression to allow for tumour growth.

## 4 DISCUSSION

CRC is characterized by the activation of oncogenic genes and inactivation of tumour suppressor genes. Colorectal tumours often metastasize to the liver, which decreases survival and reflects changes in gene and miR expression levels.^28^, ^54^, ^55^, ^56^, ^57^, ^58^ The screening of biomarkers for cancers have identified miRs as potential indicators of disease states.^28^, ^55^ *miR‐210* is a known biomarker of cancer, is involved in cancer progression and exhibits oncogenic properties in many cancers.^21^, ^59^ *miR‐210* as a potential therapeutic target is important, as it plays a role in several different biological processes such as proliferation, differentiation, apoptosis, hypoxia and angiogenesis.^21^, ^59^

There are reports of *miR‐210* target genes involved in cancer progression pathways. Other functions of increased *miR‐210* include increases in genetic instability, altering normal mitochondrial function and promoting angiogenesis and metastasis (reviewed in^26^). We propose *miR‐210* inhibition as a therapeutic approach; however, current methods to inhibit miRs in vivo are limited and with variable effectiveness.

### 4.1 New biotechnology to inhibit *miR‐210*

The PMIS inhibitor is composed of native, unmodified nucleic acid that enables the development of stably expressing cells (lentivirus) and animal models for the study of genome‐wide miR family inhibition to identify miR targets and cellular processes.^15^, ^16^, ^20^, ^60^ The PMIS inhibitor can be used to knockdown miRs during embryonic development to determine their effect on stem cells, cell proliferation and differentiation as well as developmental processes.^15^, ^16^, ^17^, ^18^, ^19^, ^20^, ^60^ The PMIS system allows researchers to finally dissect the role of miRs expressed in clusters and on multiple chromosomes. The PMIS can distinguish between and differentially inhibit miRs with only one nucleotide change in their seed sequence. The unique structure of the PMIS‐miR complex is bound by factors of the RISC, making it very stable, efficient, with a high specificity and affinity for specific miRs and is not toxic in animals and cells.^15^ The PMIS works in transgenic animals (in vivo) as well as xenograft animal models without adverse side effects, and it has great promise as a therapeutic molecule in clinical applications.

### 4.2 Therapeutic delivery

Different methods of miR inhibitor delivery must address the effectiveness and off‐target effects of treatment and include toxicity, harmful side effects and specificity. Most miR inhibitors used in therapeutic applications are antagomirs, LNAs, PNAs (peptide nucleic acids), and anti‐miR oligonucleotides.^8^, ^26^, ^61^ Because oligonucleotides must be encapsulated to be stabilized and taken up by the cell, synthetic delivery systems including lipoplexes, polyethyleneimine (PEI)‐based systems and poly(lactide‐co‐glycolide) (PLGA)‐derived nanoparticles and adaptations of nanoparticle technology have been used for targeted therapeutic delivery.^61^, ^62^, ^63^, ^64^, ^65^ Viral vectors including lentiviral vectors expressing miR sponge or antagomir have been used to knockdown miRs in cells and mice.^66^, ^67^, ^68^, ^69^, ^70^ However, these methods are not efficacious, specific and have off‐target effects.

Extracellular vesicles and exosomes as delivery methods for miRs and miR inhibitors have been shown to effectively deliver small anti‐sense oligonucleotides, miR mimics and endogenous miRs. miRs can act as mobile genetic signals both systemically and for intercellular communication.^71^ miRs contained in extracellular vesicles and exosomes can transfer information cell‐to‐cell within a tissue and from exosomes produced by many cells found in serum and saliva to other types of tissues.^72^, ^73^, ^74^, ^75^, ^76^ It has been shown that miR transport between mesenchyme and epithelial tissue during submandibular gland development is important for normal morphogenesis.^76^

### 4.3 Effective and efficacious *PMIS‐miR‐210* plasmid DNA delivery

We demonstrate using ‘naked’ PMIS plasmid DNA delivery as a safe and effective method to inhibit miRs in cells and tissues. It is well known that direct injection of plasmid DNA into tissues can express transcripts and proteins. Whether for vaccines or therapeutic treatments, plasmid DNA has been successfully injected into muscle, pancreas, brain and liver.^77^, ^78^, ^79^, ^80^, ^81^, ^82^, ^83^ However, direct injection into the blood has low efficiency and the plasmid DNA is rapidly degraded and a disadvantage of direct injection of naked plasmid DNA in some cases is that it can be degraded prior to high levels of gene expression.

We found that using highly purified supercoiled plasmid DNA (CsCL gradient isolation) at relatively low concentrations (5–10 μg) directly injected into the growing tumour produced efficient expression of the *PMIS‐miR‐210* inhibitor RNA transcript. The PMIS RNA is extremely stable in cells and not rapidly degraded.^15^ Therefore, because the PMIS molecule is stable, the cell packages *PMIS‐miR‐210* into exosomes, which can be transferred to other tumour cells and create tumour‐wide expression. This system provides a new approach as a therapeutic reagent because of its unique properties including (a) high specificity for mature miRs, (b) no toxicity, (c) stable RNA molecule, (d) in vivo efficacy, (e) does not require repeated dosing to be effective, and (f) does not require a toxic nanoparticle delivery method. Furthermore, 100% of cells need not be transfected with plasmid DNA to illicit a response. The *PMIS‐miR‐210* transcripts can be transmitted to other tumour cells by exosomes to activate apoptosis and gene expression programmes. The treated mice have normal levels of ALT, AST and bilirubin and normal liver structure, indicating no toxicity due to the administration of *PMIS‐miR‐210*.

### 4.4 *miR‐210* regulates apoptosis in colorectal tumours

*miR‐210* expression is associated with cell proliferation and apoptosis. Increased *miR‐210* expression in glioblastoma multiforme cells induces proliferation and decreases apoptosis by targeting the regulator of differentiation 1 (ROD1).^84^ *miR‐210* expression inhibits apoptosis in cardiomyocyte cells by targeting *ephrin A3*, *PTP1b*, *Casp8ap2*, *ISCU* and *Sirt3*.^21^, ^85^, ^86^ We found that *miR‐210* inhibition in SW620 metastatic colorectal tumours staining for Ki67 showed a slight but significant decrease in tumour cell proliferation. Active apoptosis was observed in tumour sections when *miR‐210* was inhibited and correlates to a decrease in tumour size. Thus, while *miR‐210* expression and inhibition of apoptosis is good for heart repair, *miR‐210* inhibition by *PMIS‐miR‐210* works to activate apoptosis in colorectal cancer.

### 4.5 The role of *XIST* and *miR‐210* in colorectal cancer

lncRNA *XIST* (X‐inactive specific transcript) is a master regulator of X inactivation in mammals. The role of *XIST* in cancer is unclear as reports suggest it is a tumour‐suppressor while other reports suggest *XIST* has tumour promotion effects in several human cancers (for a review^52^). We became interested in *XIST* after profiling SW620 cells and tumours using RNA‐seq and found that upon inhibition of *miR‐210*, *XIST* was highly expressed and associated with decreased tumour growth. *XIST* was the highest expressed transcript when *miR‐210* was inhibited in both cells and tumours, thus high *XIST* expression was correlated with decreased colorectal tumour growth.

*XIST* acts as a sponge for several microRNAs, including *miR‐126*, *miR‐485*, *miR‐214* and *miR‐497*.^86^, ^87^, ^88^, ^89^ These miRs binding to *XIST* can either promote cancer^87^, ^89^ or have anticancer effects through regulating the miR target genes.^86^, ^88^ *miR‐210* has been shown to bind to *XIST* as well as the tumour suppressor genes, *APC*, *ACVR1B*, *CDK10*, *SERTAD2*, *E2F3* and *MNT* in an *miR‐210* candidate gene screen.^48^ Furthermore, inhibition of *miR‐210* induced apoptosis in hypoxic HUVEC cells.^48^ Interestingly, *XIST* has been reported to be restricted to the nucleus and is associated with the X chromosome and it is unclear how *XIST* could act as an miR sponge.^52^ Several miRs have been found in the nucleus associated with human cancers (see review^90^). *miR‐210* could shuttle between the nucleus and cytoplasm to regulate gene expression as suggested in a previous report.^48^

Our studies found that inhibition of *miR‐210* resulted in an increase in *XIST* transcripts in both the cytoplasm and nucleus of SW620 cells expressing *PMIS‐miR‐210*. The inhibition of *miR‐210* stabilizes *XIST* transcripts. We have shown that *XIST* transcripts are expressed at low levels in these cells and localized to the nucleus in SW620 cells. We propose that *miR‐210* binds to *XIST* to rapidly inactivate and degrade *XIST* transcripts in the cytoplasm and possibly in the nucleus. Therefore, *XIST* transcripts may not be observed in the cytoplasm due to miRs binding and targeting *XIST* for degradation. Thus, *miR‐210* would reduce *XIST* expression and inhibit *XIST* from acting as a tumour suppressor. We provide evidence for the regulation of *NME1*, a tumour suppressor, through *miR‐210/XIST‐*mediated epigenetic mechanism.

### 4.6 *XIST* epigenetic regulation of *NME1* expression

Bioinformatic analyses of RNA‐seq data from *PMIS‐miR‐210* SW620 cells and tumours revealed several new genes were upregulated, including the tumour suppressor *NME1*. *NME1* was identified as a gene upregulated in both *PMIS‐miR‐210* cells and tumours. NME1 is a metastasis suppressor protein with the ability to suppress the metastatic phenotype of cancer cells without affecting primary tumour growth.^42^, ^91^ *NME1* was increased in both SW620 cells and tumours when *miR‐210* was inhibited and confirmed by qPCR and Western blot analyses. In *PMIS‐miR‐210*‐injected tumours, NME1 was widely expressed in tumour sections. However, *NME1* does not contain a binding site for *miR‐210* and is not directly regulated by *miR‐210* (data not shown). Genomic analyses of the *NME1* gene found peaks for H3K27ac and H3K4me3 near the proximal promoter of *NME1*. ChIP‐seq experiments found increased levels of H3K27ac and H3K4me3 deposition at the *NME1* proximal promoter in *PMIS‐miR‐210* SW620 cells compared to controls, indicating active enhancers/promoters.^49^ *XIST* is best known for the kinetics of X‐chromosome inactivation during differentiating female embryonic stem cells (review^92^). *XIST* is thought to initiate silenced chromatin by inhibiting H3K4me3 and H3K9ac transcriptionally active markers and interacting with H3K27me3 to repress chromatin.^93^ However, after embryonic development *XIST* may be repurposed or reprogrammed especially in cancer to act as a tumour suppressor.^52^ We show that both inhibition of *miR‐210* and overexpression of *XIST* activates both H3K27ac and H3K4me3 active chromatin markers at the *NME1* proximal promoter. Therefore, the inhibition of *miR‐210* increases *XIST* transcripts, which can interact with the *NME1* locus to open the chromatin and increase *NME1* expression.

### 4.7 *PMIS‐miR‐210* is an efficient anti‐cancer therapeutic reagent

**FIGURE 11:**
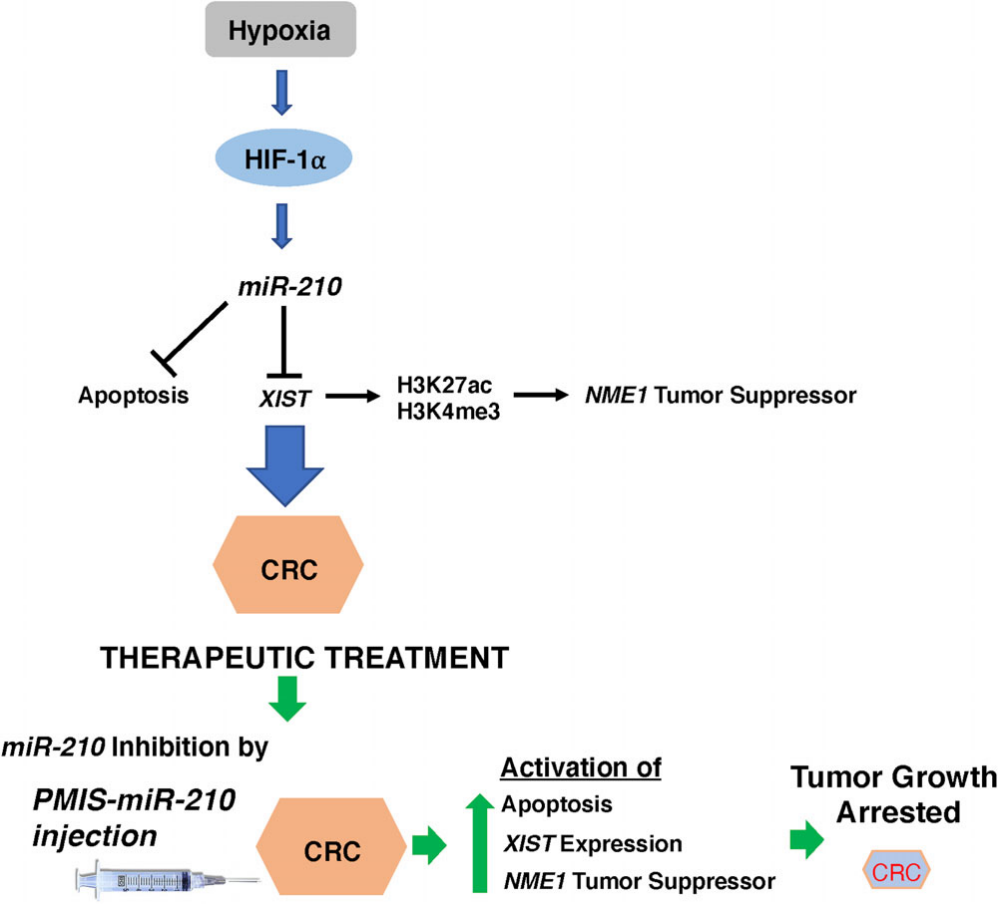
Model for the role of *miR‐210* in colorectal cancer (CRC) and arrest of tumour growth by *PMIS‐miR‐210*. Hypoxic conditions in the tumour activate *HIF1a* and *miR‐210* expression. *miR‐210* blocks apoptosis and degrades *XIST* transcripts, which cannot induce *NME1* expression through epigenetic regulation and leads to CRC. Therapeutic treatment by direct injection of plasmid DNA expressing *PMIS‐miR‐210* activates apoptosis, *XIST* and *NME1* expression, which arrests CRC tumour growth.

*miR‐210* is aberrantly expressed in multiple cancers and a previous study identified several *miR‐210* directly and indirectly regulated cellular processes associated with hypoxia and cancer.^48^ In 293 cells, *miR‐210* has been shown to regulate the genes modulating the cell cycle, differentiation, development, membrane trafficking, migration/adhesion and DNA binding.^48^ A model for the proposed function of *miR‐210* in colorectal cancer is shown in Figure 11. We used the *PMIS‐miR‐210* system to make stable cell lines and to inhibit tumour growth and identified the *NME1* tumour suppressor regulated by an *miR‐210/XIST* pathway.

To advance the potential for treatment of cancer, we directly injected naked *PMIS‐miR‐210* plasmid DNA into the tumour. We used this treatment as the FDA has approved the use of plasmid DNA previously for the treatment of diseases. However, the use of PLGA and PEI nanoparticles are not approved for gene therapy and in our experiments promote cell toxicity. We demonstrate efficient uptake of the plasmid DNA by the tumour cells and further expression of the PMIS molecule in tumour cells by extracellular vesicle transfer. These results are highly significant, and we have shown the efficacy of our treatments compared to capecitabine treatments. We are currently working for approval of the use of PMIS plasmid DNA in the treatment of several diseases and tissue regeneration. This technology will greatly advance our use of RNA therapeutics.

## 5 CONCLUSIONS

The PMIS system to effectively inhibit aberrantly expressed miRs in cancer and other diseases offers an alternative therapeutic approach. The PMIS is specific with no off‐target effects, stable and non‐toxic to cells. It is easy to administer to tumours, efficacious and safe. Because of engineered PMIS stability, it can be packaged by cells into extracellular vesicles and transferred to other cells within the tumour to effectively inhibit miRs through the tumour. These are all properties lacking in current oligonucleotide therapies. The PMIS has great promise for gene therapy and can target any overexpressed miR in multiple cancers.

## CONFLICT OF INTEREST

Brad A. Amendt is the CSO of NaturemiRI, LLC. The remaining authors declare no conflict of interest.

## Supporting information



Supporting Figure S1 DLD1 and Colo320 colorectal tumour cells express *miR‐210*. Both DLD1 and Colo320 tumour cells were analyzed for *miR‐210*, *XIST* and *NME1* expression levels and regulation by *PMIS‐miR‐210*. microRNA and gene expression was measured by qPCR in tumour cells and cells transformed with PMIS‐EV (empty vector) and PMIS‐miR‐210 (PMIS‐210). Fold activation (ΔΔ*C*_t_) and *p < .05, *N* = 3.

Supporting Figure S2 *PMIS‐miR‐210* treatment reduced tumour formation. Tumours were measured using a caliper at the time of DNA injection and after 1 week. The size was recorded by measuring the length and width (mm) of the tumour.

Supporting Figure S3 *PMIS‐miR‐210* treatments did not affect liver function. Treated and untreated mice were evaluated for liver enzymes after treatments. Aspartate transferase (AST) and alanine aminotransferase (ALT) levels in the blood were measured as a readout of lever function and disease.

Supporting Figure S4 Cell‐to‐cell transfer of *PMIS‐miR‐210* by extracellular vesicles. (A) Extracellular vesicles (ECVs) isolated from SW620 and 293 cells with and without *PMIS‐miR‐210* expression were applied to SW620 cells to determine if endogenous *miR‐210* transcript levels were affected using qPCR. ECVs from SW620 (100 μl) and 293 cells (100 μl = low and 2× = 200 μl) containing *PMIS‐miR‐210* effectively reduced *miR‐210* transcript levels. (B) ECVs from cells, as in panel A, were applied to SW620 cells and *Nme1* and *Brk1* transcript levels were measured by qPCR. *PMIS‐miR‐210* from ECVs increased the expression of *Nme1* in both SW620 and 293 cells but did not affect *Brk1* transcripts. (C) ECVs from cells, as in panel C, were applied to SW620 cells and *FGFRL1* and *XIST* transcripts were both increased when the source of ECVs came from *PMIS‐miR‐210*. *p < .05; **p < .01

Supporting Figure S5 Cell‐to‐cell transfer of PMIS‐miR‐210 inhibited by neticonazole. Donor cells in the transwell experiments were first treated with the EV inhibitor neticonazole for 48 h before the assay. Transwell experiments were performed as in Figure 9D. (A) *PMIS‐miR‐210* levels were not detected in recipient cells at the different ratios of donor cells to recipient cells. *miR‐210* levels were also unaffected. As controls, the SW620 *PMIS‐miR‐210*‐treated cells expressed *PMIS‐miR‐210* and inhibited *miR‐210* levels. (B) *XIST* levels were also unaffected after donor cell treatments in the recipient cells. *p < .05, *N* = 3

## Data Availability

All data are available in the main text and RNA‐seq data are available upon request. All PMIS constructs are available for research purposes.
